# PCBP2 Regulates p16^INK4a^ ‐Dependent Cellular Senescence in Response to Iron

**DOI:** 10.1111/acel.70283

**Published:** 2025-11-11

**Authors:** Shaoyang Yan, Lili Lu, Yuwei Wu, Zewen Du, Ebin Johny, Partha Dutta, Gang Li

**Affiliations:** ^1^ Aging Institute University of Pittsburgh Pittsburgh Pennsylvania USA; ^2^ School of Medicine Tsinghua University Beijing People's Republic of China; ^3^ Department of Cardiology The First Hospital of Hebei Medical University Shijiazhuang China; ^4^ Center for Pulmonary Vascular Biology and Medicine, Pittsburgh Heart, Lung, Blood, and Vascular Medicine Institute University of Pittsburgh School of Medicine and University of Pittsburgh Medical Center Pittsburgh Pennsylvania USA; ^5^ Vascular Medicine Institute University of Pittsburgh School of Medicine and University of Pittsburgh Medical Center, Pennsylvania Pittsburgh USA; ^6^ Department of Medicine, Division of Cardiology University of Pittsburgh Medical Center Pittsburgh Pennsylvania USA

## Abstract

Recently, we have identified *rs1333046* as one of the candidate functional single nucleotide polymorphisms (fSNPs) on the atherosclerosis‐associated CDKN2A/B locus. However, how *rs1333046* influences the pathogenesis of and susceptibility to atherosclerosis is unknown. In this work, we demonstrate that *rs1333046* is part of a *cis*‐regulatory element (*cis‐*RE) that regulates p16^INK4a^ and p16^INK4a^‐dependent cellular senescence in human endothelial cells (ECs). This is achieved by recruiting poly(rC)‐binding protein 2 (PCBP2), a member of the poly‐cytosine binding protein family. We also reveal that PCBP2 is an upstream regulator of CD40, which regulates the expression of senescence‐associated secretory phenotype (SASP) genes through NF‐κB signaling. Moreover, consistent with PCBP2 being an iron chaperone, we discover that iron can induce cellular senescence by regulating both p16^INK4a^ and CD40‐mediated SASP gene expression through PCBP2. Notably, iron dynamically regulates p16^INK4a^ expression by altering the binding of PCBP2 to *rs1333046*. In addition, reducing intracellular labile iron by overexpressing both iron storage protein ferritin light chain (FTL) and iron exporter ferroportin 1 (FPN1) in ECs suppresses cellular senescence, and overexpression of PCBP2 in both FTL‐ and FPN1‐overexpressing cells restores cellular senescence. Thus, our studies suggest that iron could be a potential environmental factor regulating atherosclerosis‐associated cellular senescence, and this is achieved by modulating PCBP2‐dependent p16^INK4a^ and CD40 expression. This study shows the mechanism by which iron affects the pathology of atherosclerosis.

## Graphical abstract

PCBP2 induces cellular senescence by modulating the expression of p16^INK4a^ and CD40 in response to iron. PCBP2 activates p16^INK4a^ via the functional SNP *rs1333046* on the atherosclerosis‐associated CDKN2A/B locus.

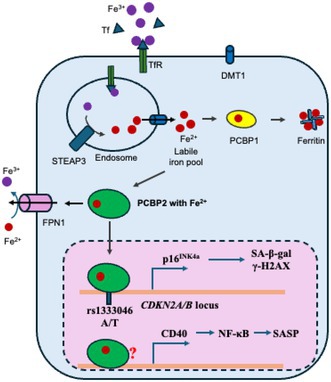

## 1 Introduction

Atherosclerosis is an age‐related disease, as its clinical incidence rises exponentially when we age. This suggests that aging could be the risk factor for atherosclerosis (Li et al. 2021; Lopez‐Otin et al. 2013; Aunan et al. 2016; Niccoli and Partridge 2012). Among all the hallmarks that contribute to aging, cellular senescence is one of the most important factors for aging and age‐related pathologies (Lopez‐Otin et al. 2023).

Cellular senescence is defined as the irreversible arrest of the cell cycle. As a result, senescent cells are characterized by an enlarged and flattened appearance as well as by many biochemical changes, including increased expression of p16^INK4a^, elevated senescence‐associated β‐galactosidase activity (SA‐β‐gal), and accumulation of phosphorylated H2A histone family member X (γ‐H2AX) (Sikora et al. 2016). In addition, senescent cells exhibit a senescence‐associated secretory phenotype (SASP), producing various pro‐inflammatory and adhesion molecules, such as IL‐6, IL‐1β, VCAM‐1 and ICAM‐1, as well as different growth factors and proteases (Saul et al. 2022). A combination of all these factors is now being used to reliably label senescent cells as no single marker has yet been identified to label these cells (Wang and Dreesen 2018). Cellular senescence can be induced either by telomere shortening that occurs during cell proliferation, or by various stresses, including DNA damage, oxidative stress, and oncogene expression (Lopez‐Vicente et al. 2009). The former is recognized as replicative senescence and the latter as premature senescence. Both types of senescence can be regulated by the p16^INK4a^ pathway (Kumari and Jat 2021; Liu and Wan 2019; Mijit et al. 2020). However, regulation of atherosclerosis‐associated senescence is largely unknown.

Genome‐wide association studies (GWAS) have identified a strong association of the locus for the cyclin‐dependent kinase inhibitor 2A/B (CDKN2A/B) with a multitude of age‐related diseases, including atherosclerosis and its related complications (Hannou et al. 2015). The CDKN2A/B locus, spanning a 200‐kb region, encodes two closely related genes *CDKN2A* and *CDKN2B* as well as antisense noncoding RNA in the *INK4* locus (ANRIL). *CDKN2A* encodes two important tumor suppressor proteins, p16^INK4a^ and p14^ARF^, while *CDKN2B* encodes another tumor suppressor protein p15^INK4b^. Among these gene products, p16^INK4a^ is both a known activator and marker of cellular senescence. It contributes to the pathogenesis of various age‐related diseases. This is because genetic or pharmacological depletion of p16^INK4a^‐positive cells in various mouse models delays the onset of age‐related diseases and extends the lifespan (Baker et al. 2011, 2016; Dang et al. 2020; Hickson et al. 2019). However, how p16^INK4a^ is activated during cellular senescence, especially in the context of atherosclerosis‐associated senescence, is so far unexplored.

Iron is an essential element that plays an important role in cellular metabolism. In the human body, iron enters the cell mainly through clathrin‐mediated endocytosis (CME), facilitated by transferrin (Tf), an iron‐binding protein. Ferric (Fe^3+^)‐loaded Tf binds to Tf receptor 1 (TfR1), and transfers Fe^3+^ into the cell via membrane internalization. Under acidic conditions, Fe^3+^ is released from Tf and reduced to the ferrous (Fe^2+^) form by six‐transmembrane epithelial antigen of prostate 3 (STEAP3). Then, Fe^2+^ enters the cytosol via divalent metal transporter 1 (DMT1) (Anderson and Frazer 2017; Peng et al. 2021). To prevent iron‐induced toxicity in the cytosol, overloaded iron must be either stored in ferritin, an iron cage made up of two functionally distinct proteins, ferritin heavy chain (FTH) and ferritin light chain (FTL) (Kotla et al. 2022; Garate and Nunez 2000), or exported out of cells through ferroportin 1 (FPN1) (Ward and Kaplan 2012; Ganz 2005), the only iron exporter discovered so far. Delivery of iron to ferritin for storage is believed to be mediated by poly(rC)‐binding protein 1 (PCBP1) (Ryu et al. 2018), whereas the transport of iron to FPN1 is mediated by PCBP2 (Yanatori et al. 2016). It has been demonstrated that iron induces ferroptosis via the Fenton reaction (Winterbourn 1995). It was also reported that iron can induce cellular senescence in human cells (Maus et al. 2023; Masaldan et al. 2018; Noh et al. 2023; Li et al. 2023). However, the underlying mechanisms of iron‐induced cellular senescence remain unclear.

PCBP2 is a member of the poly‐cytosine binding protein family which has nucleic acid‐binding activity. It is involved in the transcriptional regulation of multiple genes (Yuan et al. 2021). As a molecular chaperone of intracellular iron, PCBP2 receives and delivers iron to iron‐metabolism‐related proteins, maintaining iron homeostasis (Yanatori et al. 2020). Recently, PCBP2 was shown to regulate cell senescence in mouse embryonic fibroblasts (MEFs) (Ren et al. 2016). However, no publication has shown any functional coordination between PCBP2 and iron in regulating cellular senescence.

Based on our recently published post‐GWAS functional studies (Zhao et al. 2020; Jiang et al. 2022), we demonstrate in the present study that PCBP2 is a transcriptional regulator of p16^INK4a^, activating cellular senescence in ECs through its specific binding to a functional SNP (fSNP) *rs1333046* within the atherosclerosis‐associated CDKN2A/B locus. Moreover, consistent with our knowledge that p16^INK4a^ is not a regulator of SASP, we discovered that PCBP2 regulates SASP gene expression, at least including *IL‐6*, *IL‐1β*, and *ICAM‐1*, by modulating CD40‐induced NF‐κB signaling. Most importantly, our analysis reveals that iron can induce cellular senescence by activating p16^INK4a^, which is achieved by enhancing the binding of PCBP2 to *rs1333046*. Consistently, overexpression of both FTL and FPN1 can reduce the intracellular levels of iron, which suppresses cellular senescence by downregulating both p16^INK4a^ and CD40‐mediated SASP gene expression. Thus, we believe that our findings provide a molecular perspective for how iron induces atherosclerosis‐associated endothelial senescence. This should help us understand the mechanism underlying the contribution of iron to atherosclerosis.

## 2 Results

### 2.1 Identification of PCBP2 as a Regulatory Protein That Specifically Binds to fSNP *rs1333046*

**FIGURE 1:**
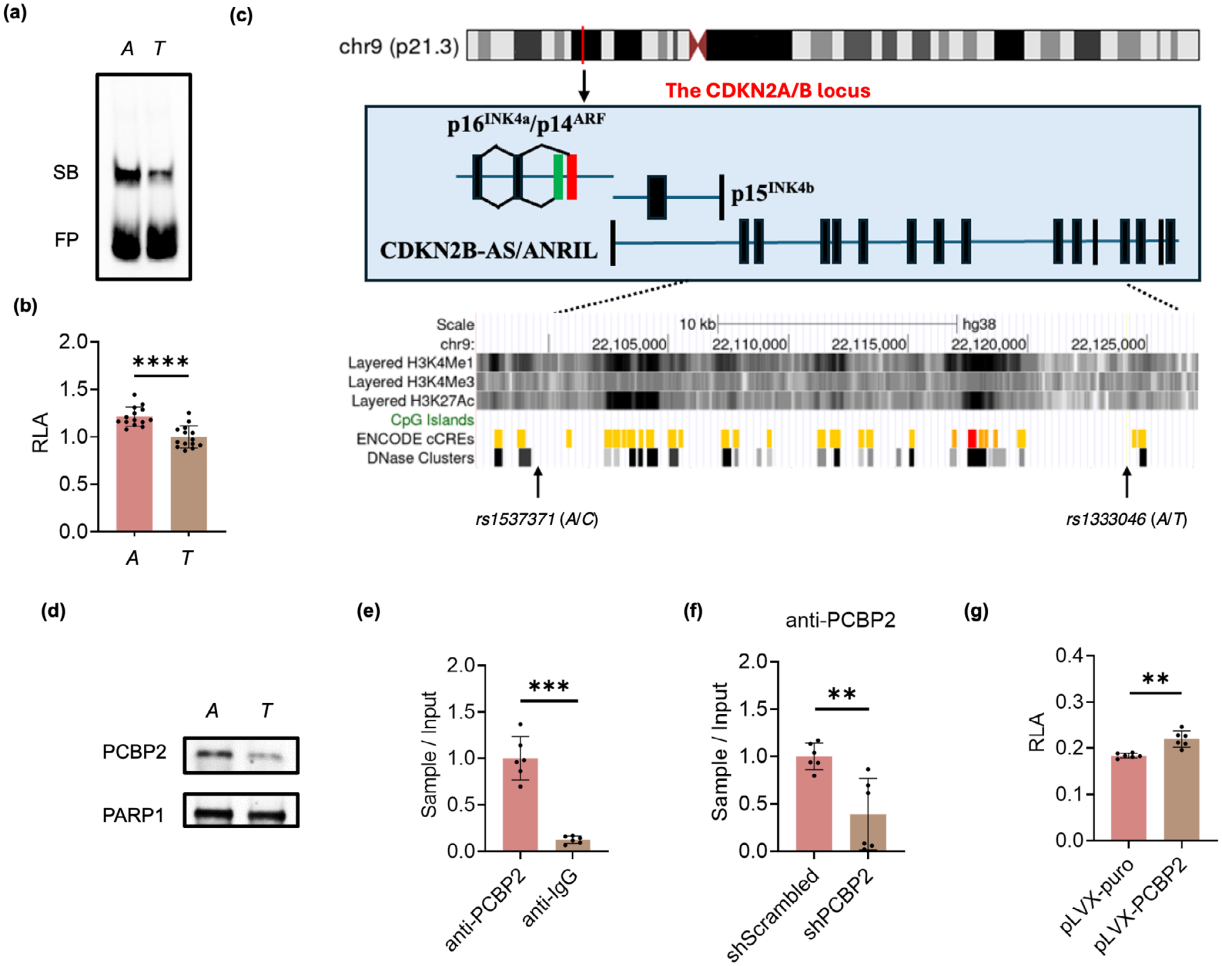
*rs1333046* is an fSNP that specifically recruits PCBP2. (a) EMSA demonstrates that *rs1333046* is an fSNP by showing allele‐imbalanced gel shifting. SB: Shifted bands; FP: Free probes. Data from EMSA represent *n* = 3 biologically independent experiments. (b) Luciferase reporter assay in 293 T cells reveals that *rs1333046* is an fSNP by showing allele‐imbalanced reporter activity. RLA: Relative luciferase activity. Data represent a combination of *n* = 14 biologically independent samples. (c) Genomic view of the CDKN2A/B locus, showing the location of *rs1333046* and *rs1537371*. Genomic tracks from the ENCODE database are: (1) Three histone modification tracks for H3K4Me1, H3K4Me3, and H3K27Ac; (2) CpG islands; (3) Candidate *cis*‐regulatory elements (cCRE) from all cell types; and (4) DNase I hypersensitivity site clusters from all cell types. (d) AIDP‐Wb using nuclear extract isolated from HUVECs demonstrates an allele‐imbalanced binding of PCBP2 to *rs1333046*. Data represent *n* = 3 biologically independent experiments. (e) ChIP assay using p10 HUVECs indicates a specific binding of PCBP2 to the DNA fragment containing fSNP *rs1333046*. Data for ChIP assay represent a combination of *n* = 3 biologically independent experiments. (f) Conditional ChIP assay demonstrates a reduced binding of PCBP2 to *rs1333046* in the shRNA‐mediated PCBP2‐knockdown p10 HUVECs. shScrambled: Scrambled shRNA control; shPCBP2: ShRNA‐mediated PCBP2 knockdown. Data for ChIP assay represent a combination of *n* = 3 biologically independent experiments. (g) Conditional luciferase reporter assay shows an increased reporter activity in the PCBP2‐overexpressing HUVECs. RLA: Relative luciferase activity; pLVX‐PCBP2: PCBP2 overexpression vector; pLVX‐puro: Control vector; anti‐PCBP2: Anti‐PCBP2 antibody; and anti‐IgG: Anti‐IgG antibody. Data represent a combination of *n* = 6 biologically independent samples. *p‐value < 0.05; **p‐value < 0.01; ***p‐value < 0.001; ****p‐value < 1 × 10^−4^.

By using Reel‐seq (regulatory element‐sequencing) (Zhao et al. 2020), we identified, in a recent study, 22 candidate fSNPs within the disease‐associated CDKN2A/B locus (Jiang et al. 2022). One of the SNPs was *rs1333046* that is in linkage disequilibrium (LD) with *rs1537371* (*R* ^2^ > 0.94), a SNP that is associated with atherosclerosis (Johnson et al. 2013; Tcheandjieu et al. 2022; Le 2021). To demonstrate that *rs1333046* is functional, electrophoresis mobility shift assay (EMSA) was performed using a nuclear extract isolated from human umbilical vein endothelial cells (HUVECs). As seen in Figure 1a, an allele‐imbalanced gel shifting was observed, with the risk allele (*A*) showing more protein binding than the non‐risk allele (*T*). Consistently, an allele‐imbalanced luciferase reporter activity was also detected, with the A allele exhibiting increased reporter activity versus the T allele in human 293 T cells (Figure 1b). To further verify that *rs1333046* is a fSNP, we also performed an *in silico* analysis using the Encyclopedia of DNA Elements (ENCODE) database. Even though *rs1333046* is aligned with neither the CpG island, candidate *cis*‐regulatory elements, nor DNase I hypersensitivity, this SNP is in a site enriched with H3K4Me1 (histone H3K4 monomethylation), H3K4Me3 (histone H3K4 trimethylation), and H3K27ac (histone H3K27 acetylation) in HUVECs (Figure 1c), suggesting that this SNP could be functional.

To further demonstrate that *rs1333046* is functional, we applied SNP‐specific DNA competition pulldown—mass spectrometry (SDCP‐MS), a DNA pulldown‐based mass spectrometry analysis (Zhao et al. 2020) and identified PCBP2 as a candidate protein that binds to *rs1333046*. Of note, PCBP2 is a poly‐cytosine binding protein as well as an iron chaperone (Yuan et al. 2021; Yanatori et al. 2020). To validate this result, we performed AIDP‐Wb (allele‐imbalanced DNA pulldown—Western blot), a technique that is used for simultaneously confirming both an fSNP and its binding protein(s) on a single Western blot (Zhao et al. 2020). An allele‐imbalanced binding of PCBP2 to *rs1333046* was detected, with the risk allele (*A*) binding more PCBP2 than the non‐risk allele (*T*) (Figure 1d). We also performed a chromatin immunoprecipitation (ChIP) assay using an antibody against human PCBP2 to detect the endogenous binding of PCBP2 to *rs1333046*. As shown in Figure 1e, an enrichment of the DNA fragments containing *rs1333046* was observed when the anti‐PCBP2 antibody was used as compared with an anti‐IgG antibody as an isotype control. In addition, a conditioned ChIP assay demonstrated a decreased binding of PCBP2 to *rs1333046* in the shRNA‐mediated PCBP2‐knockdown HUVECs (Figure 1f). A conditioned luciferase reporter assay also exhibited an upregulation of luciferase activity in the PCBP2‐overexpressing cells (Figure 1g).

Together, these data demonstrate that *rs1333046* is an fSNP, and that PCBP2 can specifically bind to *rs1333046* in an allele‐imbalanced fashion.

### 2.2 PCBP2 Regulates p16^INK4a^ Expression and Cellular Senescence in ECs

**FIGURE 2:**
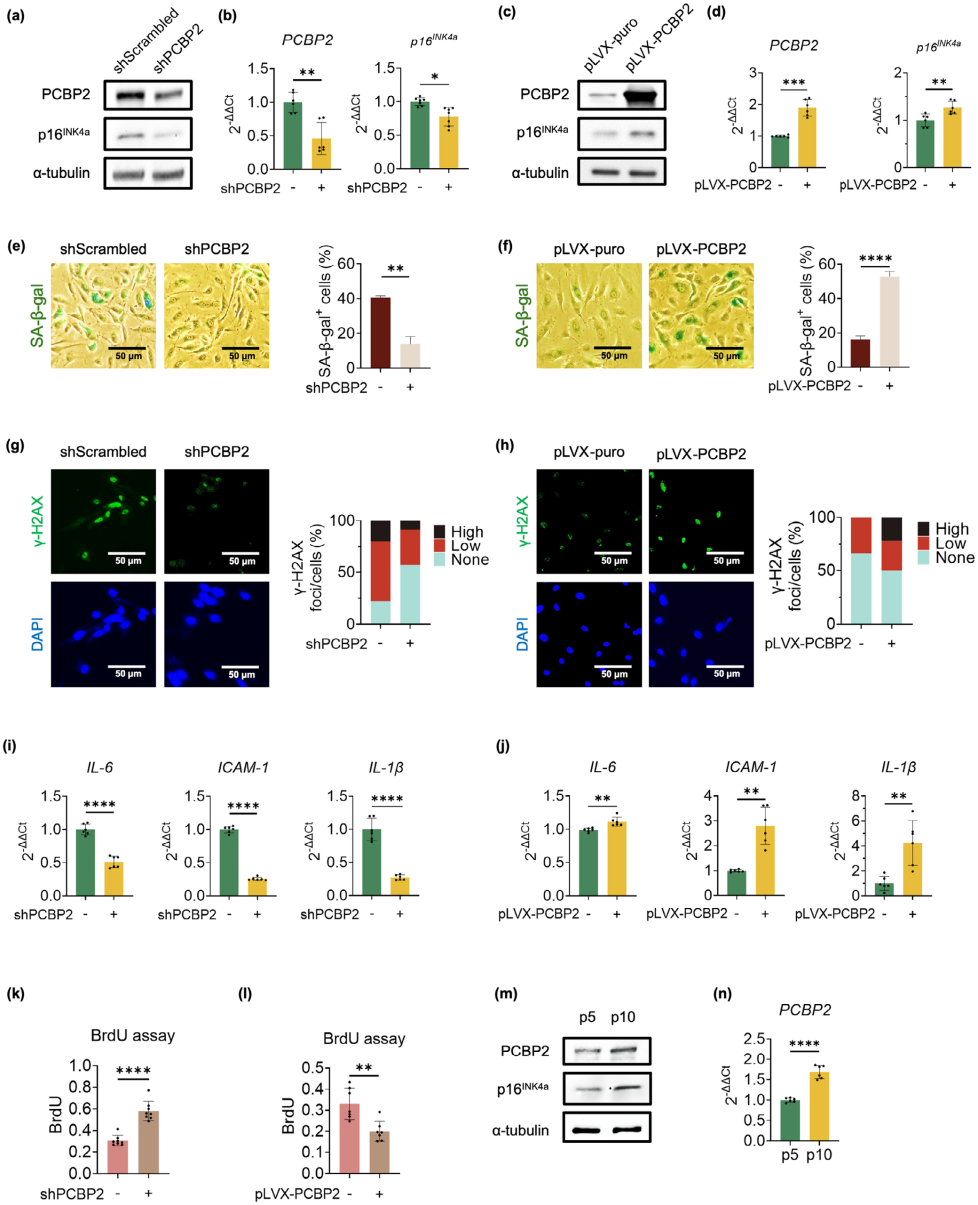
PCBP2 regulates the p16^INK4a^ expression and cellular senescence in HUVECs. (a) Western blots and (b) qPCR analyses show that shRNA‐mediated PCBP2 knockdown downregulates the p16^INK4a^ expression in p10 HUVECs. α‐tubulin was used as a loading control. Data for Western blots represent *n* = 3 biologically independent samples and data for qPCRs represent a combination of *n* = 3 biologically independent samples, each performed in duplicate. (c) Western blots and (d) qPCR analyses show that overexpression of PCBP2 upregulates the p16^INK4a^ expression in p8 HUVECs. Data for Western blots represent *n* = 3 biologically independent samples and data for qPCRs represent a combination of *n* = 3 biologically independent samples, each performed in duplicate. (e, f) SA‐β‐gal staining shows a reduced and increased number of SA‐β‐gal‐positive cells in the PCBP2‐knockdown p10 and the PCBP2‐overexpressing p8 HUVECs, respectively. Quantitative plots (right) are shown for SA‐β‐gal‐positive cells (%) in each group. Data represent *n* = 3 biologically independent samples. (g, h) γ‐H2AX staining shows a decreased and an increased γ‐H2AX staining in the PCBP2‐knockdown p10 and the PCBP2‐overexpressing p8 HUVECs, respectively. Quantitative plots (right) are shown for γ‐H2AX foci/cells (%) in each group. DAPI stains the nuclei and serves as an internal control. Data represent *n* = 3 biologically independent samples. (i, j) qPCR analyses show a significantly downregulated expression of the SASP genes *IL‐6*, *ICAM‐1*, and *IL‐1β* in the shRNA‐mediated PCBP2‐knockdown p10 HUVECs and an upregulated expression in the PCBP2‐overexpressing p8 HUVECs. Data for qPCRs represent a combination of *n* = 3 biologically independent samples, each performed in duplicate. (k, l) BrdU incorporation shows a significant increase in the shRNA‐mediated PCBP2‐knockdown p10 HUVECs and a decrease in the PCBP2‐overexpressing p8 HUVECs. Data for BrdU incorporation assays represent *n* = 8 biologically independent samples. (m) Western blots and (n) qPCR analyses show passage‐dependent increased expression of PCBP2 and p16^INK4a^ in p10 compared to p5 HUVECs. Data for Western blots represent *n* = 3 biologically independent samples and data for qPCRs represent a combination of *n* = 3 biologically independent samples, each performed in duplicate. shScrambled: Scrambled shRNA control; shPCBP2: ShRNA‐mediated PCBP2 knockdown; pLVX‐PCBP2: PCBP2 overexpression vector; pLVX‐puro: Control vector. *p‐value < 0.05; **p‐value < 0.01; ***p‐value < 0.001; ****p‐value < 1 × 10^−4^.

The binding of PCBP2 to *rs1333046* suggests that PCBP2 could be a transcriptional regulator of genes within the CDKN2A/B locus encoding p14^ARF^, p15^INK4b^, and p16^INK4a^. As *rs1333046* is associated with age‐related atherosclerosis (Tcheandjieu et al. 2022) and p16^INK4a^ is a known activator and marker of cellular senescence (Wu et al. 2023), we tested the possibility that PCBP2‐mediated regulation of p16^INK4a^ expression might have important implications in endothelial senescence. Compared to the scrambled shRNA controls, shRNA‐mediated PCBP2 knockdown significantly reduced p16^INK4a^ expression at both protein and mRNA levels (Figure 2a,b). Conversely, overexpression of PCBP2 using a lentiviral vector pLVX upregulated p16^INK4a^ expression as shown in Figure 2c,d. These data suggest that PCBP2 is a transcriptional activator of p16^INK4a^. At the same time, our Western blot analysis also demonstrated that both p14^ARF^ and p15^INK4b^ were regulated by PCBP2 (Figure S1).

To test if PCBP2 is a regulator of p16^INK4a^‐dependent cellular senescence, late passage 10 (p10) HUVECs were stained by the two senescence markers SA‐β‐gal and γ‐H2AX. As shown in Figure 2e,g, a significant decrease in the number of cells positive for both SA‐β‐gal and γ‐H2AX staining in the shRNA‐mediated PCBP2 knockdown ECs compared to the scrambled shRNA controls was observed. Conversely, a significant increase in the number of these cells was detected when PCBP2 was overexpressed in p8 HUVECs (Figure 2f,h). In addition, the expression of SASP genes *IL‐6*, *ICAM‐1*, and *IL‐1β*, was also measured and, as expected, down‐ and up‐regulation of these three genes in the shRNA‐mediated PCBP2‐knockdown (Figure 2i) and the PCBP2‐overexpressing cells (Figure 2j) were detected, respectively. Since cellular senescence is typically characterized by cell cycle arrest, we also demonstrated that downregulation of PCBP2 increases BrdU incorporation and vice versa (Figure 2k,l), suggesting that PCBP2 is indeed implicated in cell cycle arrest. Moreover, both Western blot (Figure 2m) and qPCR analysis (Figure 2n) showed a passage‐dependent expression of PCBP2 in p10 versus p5 HUVECs, further suggesting that PCBP2 is involved in replicative senescence.

**FIGURE 3:**
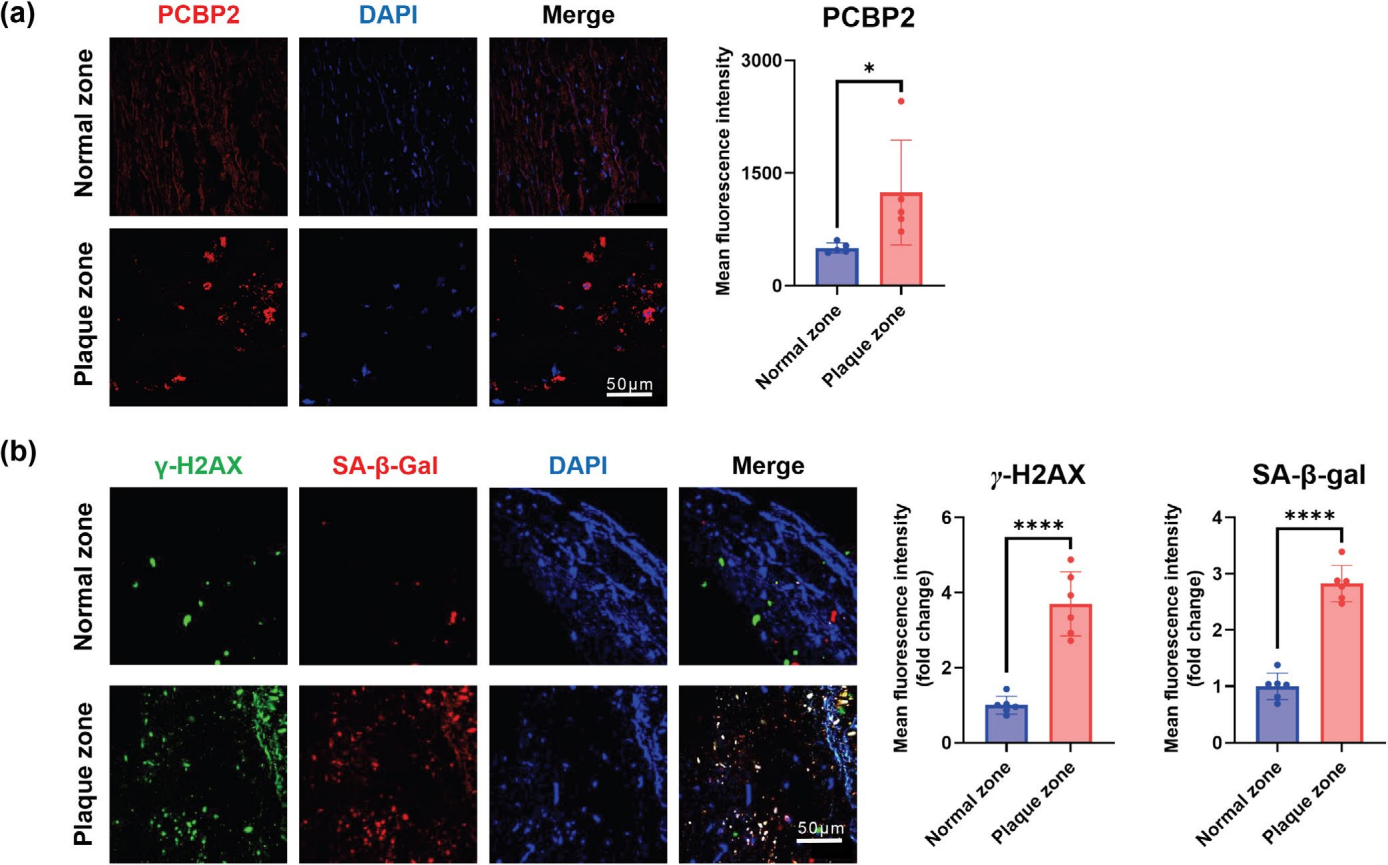
Significant induction of PCBP2 and SA‐β‐gal and γ‐H2AX staining in plaque zones versus normal‐appearing zones in carotid artery atherosclerosis samples. Immunocytochemical staining with antibodies specifically against (a) PCBP2 as well as (b) SA‐β‐gal and γ‐H2AX in plaque and normal‐appearing zones from patients with carotid artery atherosclerosis. DAPI stains the nuclei and serves as an internal control. The data were presented in median ± interquartile range, and *p*‐values were calculated with the nonparametric Mann–Whitney test for pairwise comparison. Atherosclerotic plaques were obtained from patients undergoing carotid endarterectomy at the Department of Surgery at UPMC Presbyterian Hospital. The use of human materials was approved by the University of Pittsburgh (Institutional Review Board no. STUDY18100138 and STUDY19040357), and written informed consent was obtained from all individuals before operative procedures. *p‐value < 0.05; **p‐value < 0.01; ***p‐value < 0.001; ****p‐value < 1 × 10^−4^.

Together with the data presented in Figure 1, our results indicate that PCBP2 is an activator of p16^INK4a^, regulating p16^INK4a^‐dependent cellular senescence via the atherosclerosis‐associated fSNP *rs1333046*. Consistent with this finding, we found a significant induction of PCBP2 staining (Figure 3a), along with a significant increase in staining of SA‐β‐gal and γ‐H2AX in plaque compared to normal‐appearing zones obtained from patients with carotid artery atherosclerosis. (Figure 3b). This result aligns with previous publications showing an induction of p16^INK4a^ in atherosclerotic lesions (Holdt et al. 2011; Jiang et al. 2022).

### 2.3 PCBP2 Regulates Cellular Senescence Partially via p16^INK4a^

**FIGURE 4:**
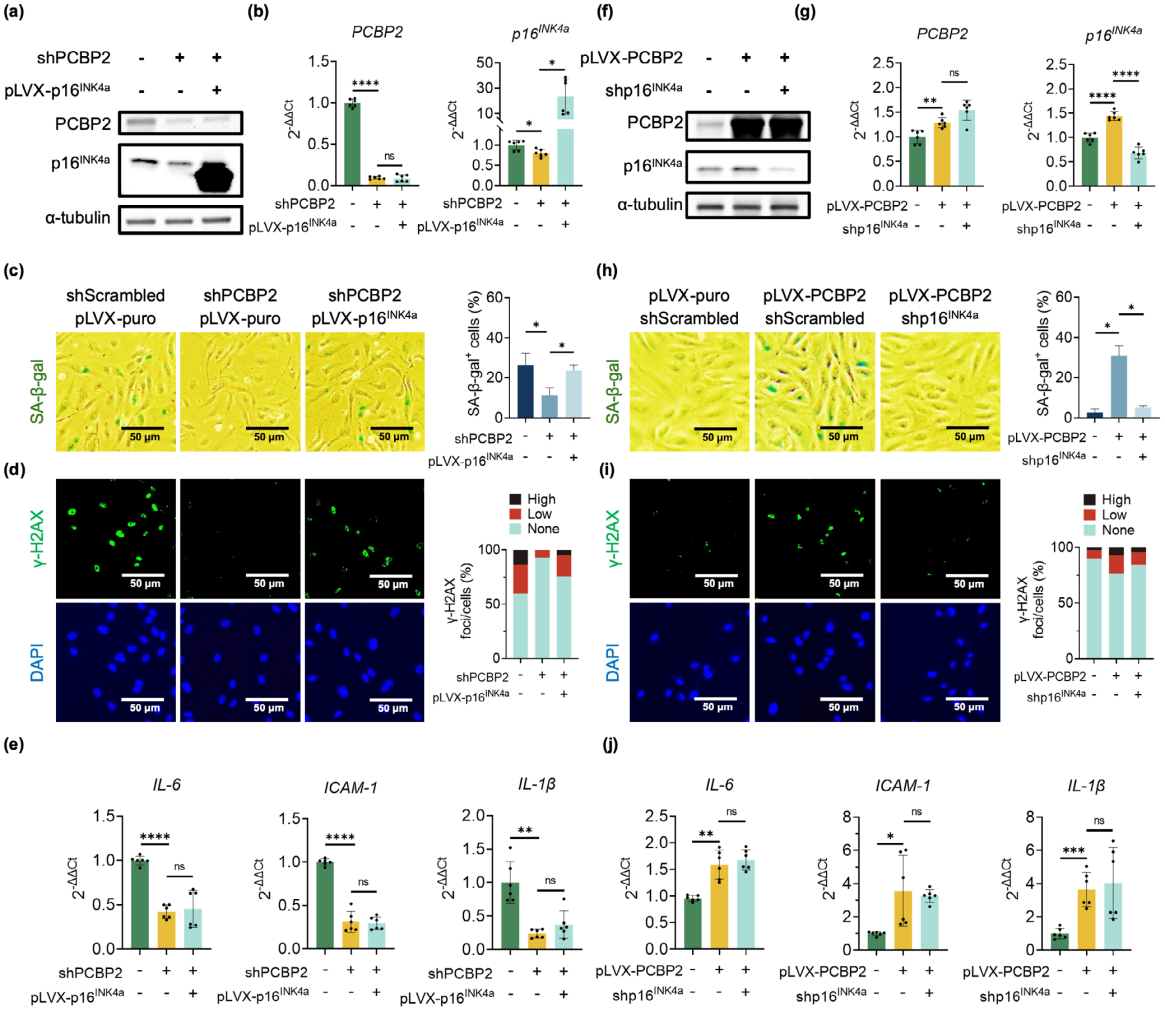
PCBP2 regulates cellular senescence by activating the p16^INK4a^ expression in HUVECs. (a) Western blots and (b) qPCR analyses show overexpression of p16^INK4a^ in shRNA‐mediated PCBP2 knockdown p10 HUVECs. Data for Western blots represent *n* = 3 biologically independent samples and data for qPCRs represent a combination of *n* = 3 biologically independent samples, each performed in duplicate. (c) SA‐β‐gal and (d) γ‐H2AX staining show that knockdown of PCBP2 in p10 HUVECs inhibits cellular senescence; ectopic overexpression of p16^INK4a^ in these cells restores senescence. DAPI stains the nuclei and serves as an internal control. Quantitative plots (right) are shown for SA‐β‐gal‐positive cells (%) and γ‐H2AX foci/cells (%). Data represent *n* = 3 biologically independent samples. (e) qPCR analyses show a decreased expression of the three SASP genes *IL‐6*, *ICAM‐1*, and *IL‐1β* in the PCBP2‐knockdown p10 HUVECs, but expressions of these three SASP genes remained unchanged when p16^INK4a^ was overexpressed in these cells. Data for qPCR analysis represent a combination of *n* = 3 biologically independent samples, each performed in duplicate. (f) Western blots and (g) qPCR analyses show downregulation of p16^INK4a^ in the PCBP2 overexpressing p8 HUVECs. Data for Western blots represent *n* = 3 biologically independent samples and data for qPCRs represent a combination of *n* = 3 biologically independent samples, each performed in duplicate. (h) SA‐β‐gal and (i). γ‐H2AX staining show that overexpression of PCBP2 induces cellular senescence, which is rescued by shRNA‐mediated p16^INK4a^ knockdown in p8 HUVECs. DAPI stains the nuclei and serves as an internal control. Quantitative plots (right) are shown for SA‐β‐gal‐positive cells (%) and γ‐H2AX foci/cells (%). Data represent *n* = 3 biologically independent samples. (j) qPCR analyses show an increased expression of the three SASP genes *IL‐6*, *ICAM‐1*, and *IL‐1β* in the PCBP2‐overexpressing p8 HUVECs, but expression of these three SASP genes remained unchanged when p16^INK4a^ was downregulated in these cells. Data for qPCR analysis represent a combination of *n* = 3 biologically independent samples, each performed in duplicate. shScrambled: Scrambled shRNA control; shPCBP2: shRNA‐mediated PCBP2 knockdown; pLVX‐PCBP2: PCBP2 overexpression vector; shp16^INK4a^: shRNA‐mediated p16^INK4a^ knockdown; pLVX‐p16^INK4a^: P16^INK4a^ overexpression vector; pLVX‐puro: Control vector. *p‐value < 0.05; **p‐value < 0.01; ***p‐value < 0.001; ****p‐value < 1 × 10^−4^.

To demonstrate that PCBP2 indeed regulates p16^INK4a^‐dependent cellular senescence, we overexpressed p16^INK4a^ in the shRNA‐mediated PCBP2‐knockdown ECs. Knockdown of PCBP2 resulted in a decreased expression of p16^INK4a^ (Figure 4a,b, left and middle lanes) and a reduction in cellular senescence, as visualized by a decreased staining of both SA‐β‐gal and γ‐H2AX (Figure 4c,d, left and middle columns) and a reduced expression of the three SASP genes *IL‐6*, *ICAM‐1*, and *IL‐1β* (Figure 4e, left and middle lanes). However, when p16^INK4a^ was overexpressed in the shRNA‐mediated PCBP2‐knockdown ECs, even though we observed an expected recovery of both SA‐β‐gal and γ‐H2AX staining (Figure 4a,b, right lanes) (Figure 4c,d, right columns), the decreased expression levels of SASP genes were not recovered (Figure 4e, right lanes). These data indicate that the expression of the analyzed SASP genes (*IL‐6*, *ICAM‐1*, and *IL‐1β*) is not regulated by p16^INK4a^.

To further demonstrate that PCBP2 regulates p16^INK4a^‐dependent cellular senescence, we downregulated p16^INK4a^ in the PCBP2‐overexpressing human ECs. This resulted in the overexpression of PCBP2 in human ECs, leading to an upregulation of p16^INK4a^ expression (Figure 4f,g, left and middle) and an induction of cellular senescence (Figure 4h–j, left and middle columns/lanes). However, shRNA‐mediated p16^INK4a^ knockdown in the PCBP2‐overexpressing ECs could only restore the SA‐β‐gal and γ‐H2AX staining to the levels seen in controls (Figure 4h,i, right columns) but not SASP gene expression (Figure 4j, right lanes).

Taken together, these results confirm that p16^INK4a^ acts as a downstream mediator of PCBP2 when regulating cellular senescence. However, regulation of cellular senescence by PCBP2 is only partially dependent on p16^INK4a^, since p16^INK4a^ does not regulate PCBP2‐mediated SASP gene expression. This is consistent with previous studies demonstrating that p16^INK4a^ is not a regulator of SASP gene expression (Coppe et al. 2011; Rodier et al. 2009).

### 2.4 PCBP2 Regulates SASP Gene Expression by Activating the CD40—NF‐κB Signaling Pathway

**FIGURE 5:**
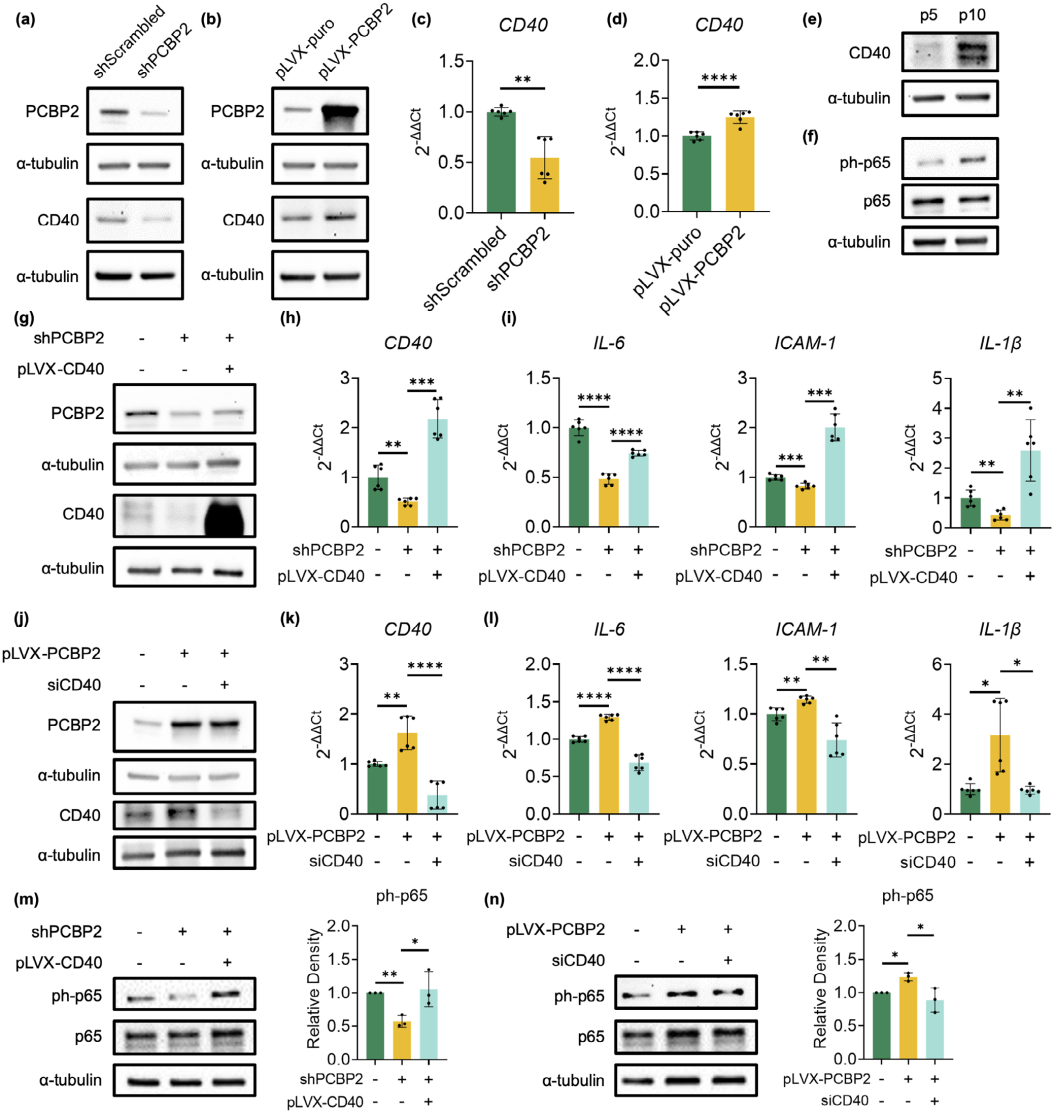
PCBP2 regulates the expression of SASP genes by activating the CD40–NF‐κB signaling pathway. (a–d) Western blots and qPCR analyses show that shRNA‐mediated PCBP2 knockdown in p10 HUVECs downregulates the CD40 expression, whereas overexpression of PCBP2 in p8 HUVECs upregulates the CD40 expression. Data for Western blots represent *n* = 3 biologically independent samples and data for qPCRs represent a combination of *n* = 3 biologically independent samples, each performed in duplicate. (e, f) Western blot analyses show a passage‐dependent increased expression of CD40 and phosphorylation of NF‐κB p65 (ph‐p65) in p10 HUVECs compared to p5 HUVECs. Data for Western blots represent *n* = 3 biologically independent experiments. (g) Western blots and (h) qPCR analyses show overexpression of CD40 in the shRNA‐mediated PCBP2‐knockdown ECs. Data for Western blots represent *n* = 3 biologically independent samples and data for qPCRs represent a combination of *n* = 3 biologically independent samples, each performed in duplicate. (i) qPCR analyses show that overexpression of CD40 in the shRNA‐mediated PCBP2 knockdown p10 HUVECs restores the expression of the SASP genes *IL‐6*, *ICAM‐1*, and *IL‐1β*, and overexpression of CD40 in these cells recovers the SASP gene expression. Data for qPCR analysis represent a combination of *n* = 3 biologically independent samples, each performed in duplicate. (j) Western blots and (k) qPCR analyses show a siRNA‐mediated CD40 knockdown in the PCBP2‐overexpressing p8 HUVECs. Data for Western blots represent *n* = 3 biologically independent samples and data for qPCRs represent a combination of *n* = 3 biologically independent samples, each performed in duplicate. (l) qPCR analyses show that siRNA‐mediated CD40 knockdown in the PCBP2 overexpressing p8 HUVECs downregulated the expression of the SASP genes *IL‐6*, *ICAM‐1*, and *IL‐1β*. Data for qPCR analysis represent a combination of *n* = 3 biologically independent samples, each performed in duplicate. (m) Western blot analyses show a reduced phosphorylation of NF‐κB p65 following PCBP2 knockdown, which can be reversed by overexpression of CD40 in the PCBP2‐knockdown p10 HUVECs. (n) Western blot analyses show an upregulated phosphorylation of NF‐κB p65 following PCBP2 overexpression, which can be reversed by siRNA‐mediated CD40 knockdown in the PCBP2 overexpression p8 HUVECs. Relative densities of NF‐κB p65 phosphorylation to total protein are shown in m and n. shScrambled: Scrambled shRNA control; shPCBP2: shRNA‐mediated PCBP2 knockdown; pLVX‐PCBP2: PCBP2 overexpression vector; pLVX‐CD40: CD40 overexpression vector; siCD40: siRNA‐mediated CD40 knockdown; p65: NF‐κB p65; ph‐p65: Phosphorylation of NF‐κB p65. Data for Western blots represent *n* = 3 biologically independent experiments. *p‐value < 0.05; **p‐value < 0.01; ***p‐value < 0.001; ****p‐value < 1 × 10^−4^.

Increasing evidence suggests that NF‐κB is a regulator of SASP genes (Salminen et al. 2012; Chien et al. 2011). NF‐κB can be activated by different signaling pathways (Hayden and Ghosh 2008; Rao et al. 2010; Yu et al. 2020), among which CD40‐induced NF‐κB signaling has been implicated in regulating SASP gene expression (Xu et al. 2018). According to our recent findings, increased CD40 expression can activate a set of pro‐inflammatory proteins, including IL‐6, IL‐1β, and ICAM‐1, via the NF‐κB p65 pathway (Zou et al. 2021). Moreover, CD40 has also been linked to the development of atherosclerosis (Lutgens et al. 2007; Lacy et al. 2021; Mach et al. 1998). As both PCBP2 and CD40 are implicated in regulating SASP gene expression, we hypothesize that PCBP2 may regulate SASP gene expression via activating the CD40–NF‐κB signaling pathway. To test this, both our Western blots and qPCR analyses showed that downregulation of PCBP2 in human ECs resulted in decreased CD40 expression (Figure 5a,c), and, conversely, overexpression of PCBP2 upregulated CD40 (Figure 5b,d). Moreover, consistent with PCBP2 being a passage‐dependent protein expressed more in p10 versus p5 HUVECs (Figure 2m,n), our Western blot analysis also showed a passage‐dependent expression of CD40 with an increased expression in p10 versus p5 HUVECs (Figure 5e). A passage‐dependent activation of NF‐κB p65 was observed with an increased phosphorylation at Ser536 in p10 versus p5 HUVECs (Figure 5f). Together, these data suggest that CD40 is a downstream effector regulated by PCBP2 in a passage‐dependent fashion. However, currently we do not know whether PCBP2 directly regulates CD40 or not.

To further demonstrate that CD40 serves as a downstream mediator of PCBP2 to regulate SASP gene expression, we either overexpressed CD40 in the shRNA‐mediated PCBP2‐knockdown ECs (Figure 5g,h) or, conversely, downregulated CD40 expression in the PCBP2‐overexpressing ECs (Figure 5j,k). As shown in Figure 5i,l, while overexpression of CD40 in the shRNA‐mediated PCBP2‐knockdown ECs significantly recovered the downregulated expression of the SASP genes *IL‐6*, *IL‐1β*, and *ICAM‐1*, siRNA‐mediated CD40 knockdown in the PCBP2‐overexpressing ECs reversed the upregulated expression of these SASP genes. Consistent with NF‐κB p65 being the downstream factor of CD40, while shRNA‐mediated knockdown of PCBP2 mitigated the phosphorylation of NF‐κB p65, ectopic overexpression of CD40 recovered the latter (Figure 5m). Conversely, siRNA‐mediated CD40 knockdown in the PCBP2‐overexpressing ECs reversed the phosphorylation of NF‐κB p65 (Figure 5n).

Taken together, these data suggest that PCBP2 regulates SASP gene expression via the CD40–NF‐κB signaling pathway.

### 2.5 Iron Dynamically Activates p16^INK4a^ and Induces Cellular Senescence via Enhancing the Binding of PCBP2 to *rs1333046*

**FIGURE 6:**
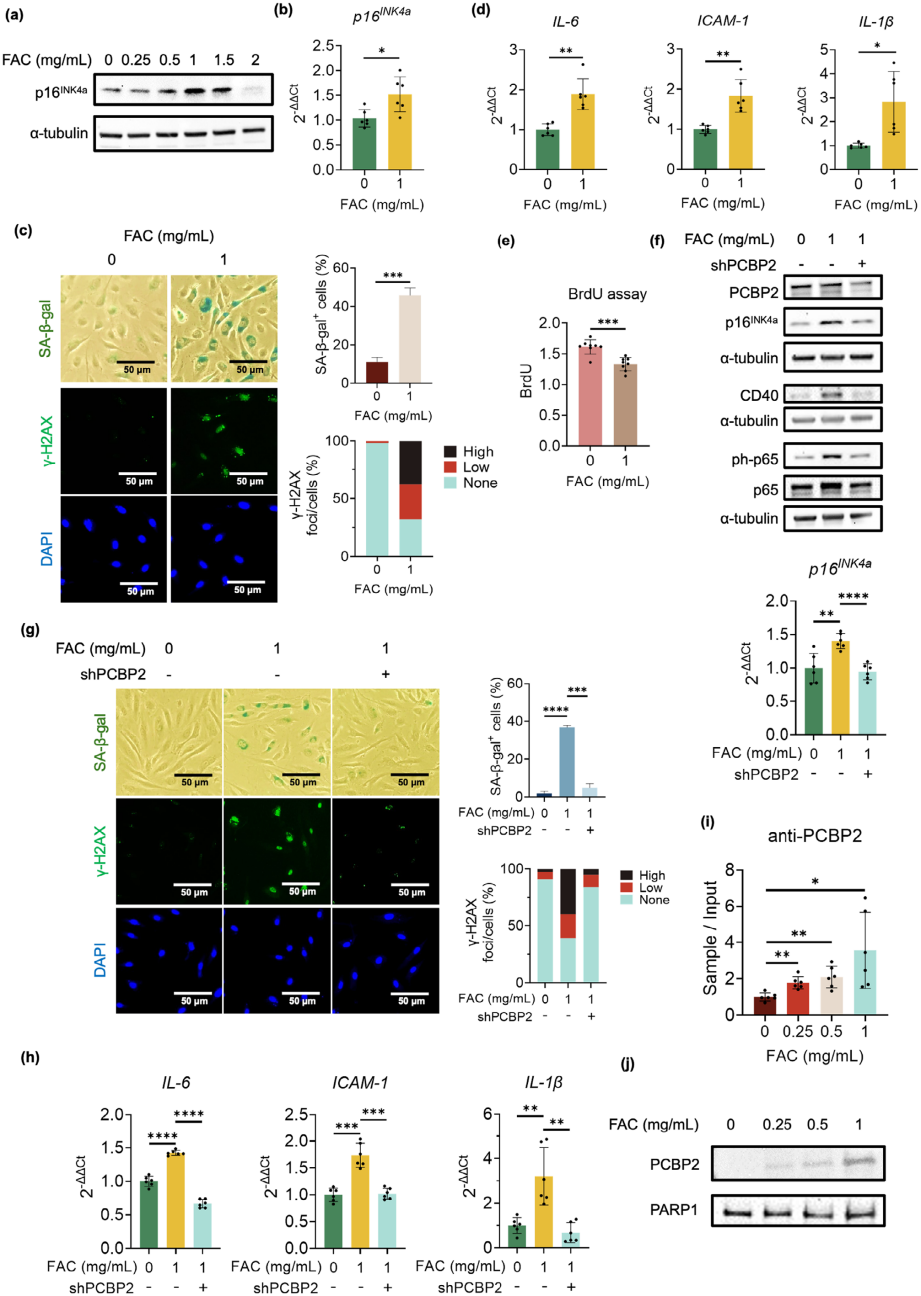
Iron activates p16^INK4a^ and cellular senescence by enhancing the binding of PCBP2 to *rs1333046*. (a) Western blots and (b) qPCR analyses demonstrate an increased p16^INK4a^ expression with increasing concentrations of FAC in ECs. Data for Western blots represent *n* = 3 biologically independent experiments and data for qPCR analyses represent a combination of *n* = 3 biologically independent samples, each performed in duplicate. (c) SA‐β‐gal (top row pictures) and γ‐H2AX (middle row pictures) staining indicate that FAC treatment induces cellular senescence in p8 HUVECs. DAPI stains the nuclei and serves as internal control. Quantitative plots (right) are shown for SA‐β‐gal‐positive cells (%) and γ‐H2AX foci/cells (%). Data for SA‐β‐gal and γ‐H2AX staining represent *n* = 3 biologically independent samples. (d) qPCR analyses demonstrate that FAC treatment induces the expression of the SASP genes *IL‐6*, *ICAM‐1*, and *IL‐1β* in p8 HUVECs. Data for qPCR analysis represent a combination of *n* = 3 biologically independent samples, each performed in duplicate. (e) BrdU incorporation assay shows a decreased BrdU incorporation in the FAC‐treated p8 HUVECs. Data for BrdU incorporation assays represent *n* = 8 biologically independent samples. (f) Western blots (top) and qPCR analyses (bottom) demonstrate that FAC treatment increases the expression of p16^INK4a^ and CD40 as well as the phosphorylation of NF‐κB p65 in p8 HUVECs, while knockdown of PCBP2 in the FAC‐treated cells reverses the expression of p16^INK4a^ and CD40 as well as the phosphorylation of NF‐κB p65 (ph‐p65). Data for Western blots represent *n* = 3 biologically independent experiments and data for qPCR analyses represent a combination of *n* = 3 biologically independent samples, each performed in duplicate. (g) SA‐β‐gal and γ‐H2AX staining indicate that FAC treatment increases cellular senescence, while knockdown of PCBP2 in the FAC‐treated p8 HUVECs suppresses cellular senescence. DAPI stains the nuclei and serves as internal control. Quantitative plots (right) are shown for SA‐β‐gal‐positive cells (%) and γ‐H2AX foci/cells (%). Data represent *n* = 3 biologically independent samples. (h) qPCR analyses demonstrate that FAC treatment increases the expression of the SASP genes *IL‐6*, *ICAM‐1*, and *IL‐1β* in p8 HUVECs, and knockdown of PCBP2 decreases their levels in these FAC‐treated cells. Data for qPCR analysis represent a combination of *n* = 3 biologically independent samples, each performed in duplicate. (i) ChIP assays demonstrate an increased binding of PCBP2 to *rs1333046* in p8 HUVECs treated with increasing concentrations of FAC from 0, to 0.25, 0.5, and 1 mg/mL by showing an increased amount of DNA fragment containing *rs1333046* being pulled down. Data for ChIP assay represent *n* = 3 biologically independent experiments. (j) DNA pulldown‐Western blot assays demonstrate an increased binding of PCBP2 to *rs1333046* in p8 HUVECs treated with increasing concentrations of FAC from 0 to 0.25, 0.5, and 1 mg/mL by showing increased levels of PCBP2 pulled down by the DNA fragment containing *rs1333046*. Data for DNA pulldown‐Wb assay represent *n* = 3 biologically independent experiments. FAC: Ferric ammonium citrate; shPCBP2: ShPCBP2: ShRNA‐mediated PCBP2 knockdown; anti‐PCBP2: Anti‐PCBP2 antibody; p65: NF‐κB p65; ph‐p65: Phosphorylation of NF‐κB p65; *p‐value < 0.05; **p‐value < 0.01; ***p‐value < 0.001; ****p‐value < 1 × 10^−4^.

As PCBP2 functions as an iron chaperone that delivers iron to FPN1 for exporting (Yanatori et al. 2016, 2020) and iron was involved in regulating cellular senescence and atherosclerosis (Maus et al. 2023; Masaldan et al. 2018; Crescenzi et al. 2023; Luo et al. 2022; Ma et al. 2022), we wondered whether iron could regulate cellular senescence by altering the morphology of PCBP2, therefore, modulating the binding of PCBP2 to the atherosclerosis‐associated fSNP *rs1333046*. To answer this question, ECs were first treated with FAC at different concentrations for 24 h. Gradually increasing the concentration of FAC from 0.25 to 1 mg/mL increased the expression of p16^INK4a^ (Figure 6a). However, at 2 mg/mL, FAC suppressed p16^INK4a^ expression (Figure 6a), suggesting that iron can dynamically regulate p16^INK4a^ expression. The activation of p16^INK4a^ was confirmed by qPCR analysis using ECs treated with 1 mg/mL FAC (Figure 6b), indicating a transcriptional regulation of p16^INK4a^. Consistent with the increased expression of p16^INK4a^, induction of cellular senescence under the same 1 mg/mL FAC treatment was visualized by increased SA‐β‐gal and γ‐H2AX staining (Figure 6c), upregulated SASP gene expression (Figure 6d), and reduced BrdU incorporation (Figure 6e). Together, these data demonstrate that iron can regulate p16^INK4a^ expression and induce cellular senescence in human ECs.

To confirm that iron regulates p16^INK4a^ expression as well as cellular senescence via PCBP2, we downregulated PCBP2 expression in ECs treated with 1 mg/mL FAC. While FAC treatment upregulated p16^INK4a^ expression of protein and mRNA levels (Figure 6f, upper and lower, left and middle lanes), as well as inducing cellular senescence (Figure 6g,h, left and middle columns/lanes), downregulation of PCBP2 in the FAC‐treated ECs restored the p16^INK4a^ expression and inhibited cellular senescence (Figure 6f–h, middle and right lanes/columns). Thus, these data demonstrate that iron can upregulate cellular senescence through PCBP2.

Consistent with our observation that PCBP2 regulates SASP gene expression via the CD40 – NF‐κB signaling pathway as shown in Figure 5, we also observed that FAC treatment increased the levels of CD40 as well as causing phosphorylation of NF‐κB p65 (Figure 6f, left and middle lanes), each of which was suppressed by shRNA‐mediated PCBP2 knockdown (Figure 6f, right lane).

To further demonstrate that iron can activate p16^INK4a^‐dependent cellular senescence by modulating the binding of PCBP2 to *rs1333046*, presumably by inducing a morphological change of PCBP2, we conducted both a ChIP assay and a DNA pulldown – Western blot analysis using the ECs treated with FAC at concentrations of 0, 0.25, 0.5, and 1 mg/mL. In both assays, with increasing concentrations of FAC, more PCBP2 was bound to the DNA fragment containing the fSNP *rs1333046* (Figure 6i,j). As ChIP assays detect endogenous protein‐DNA binding while DNA pulldown‐Western blots detect exogenous binding, our results suggest that iron can induce the binding of PCBP2 to *rs1333046*.

In addition, iron has also been reported to induce ferroptosis, a cell death induced by lipid peroxidation (Winterbourn 1995). Therefore, we tested if treating ECs with FAC also results in ferroptosis. Our preliminary results showed that very little oxidized lipid was detected in ECs treated with a concentration less than 1 mg/mL FAC. However, oxidized lipids were observed when ECs were treated with 2 mg/mL FAC (Figure S2), at which p16^INK4a^ expression was suppressed (Figure 6a). While these data need to be confirmed, it appears that low iron levels can induce cellular senescence, whereas high levels activate ferroptosis.

Taken together, these data demonstrate that iron can dynamically activate p16^INK4a^ and induce cellular senescence by enhancing the binding of PCBP2 to *rs1333046*.

### 2.6 Overexpression of Ferritin and FPN1 Mitigates Iron‐Induced p16^INK4a^ ‐Dependent Cellular Senescence

**FIGURE 7:**
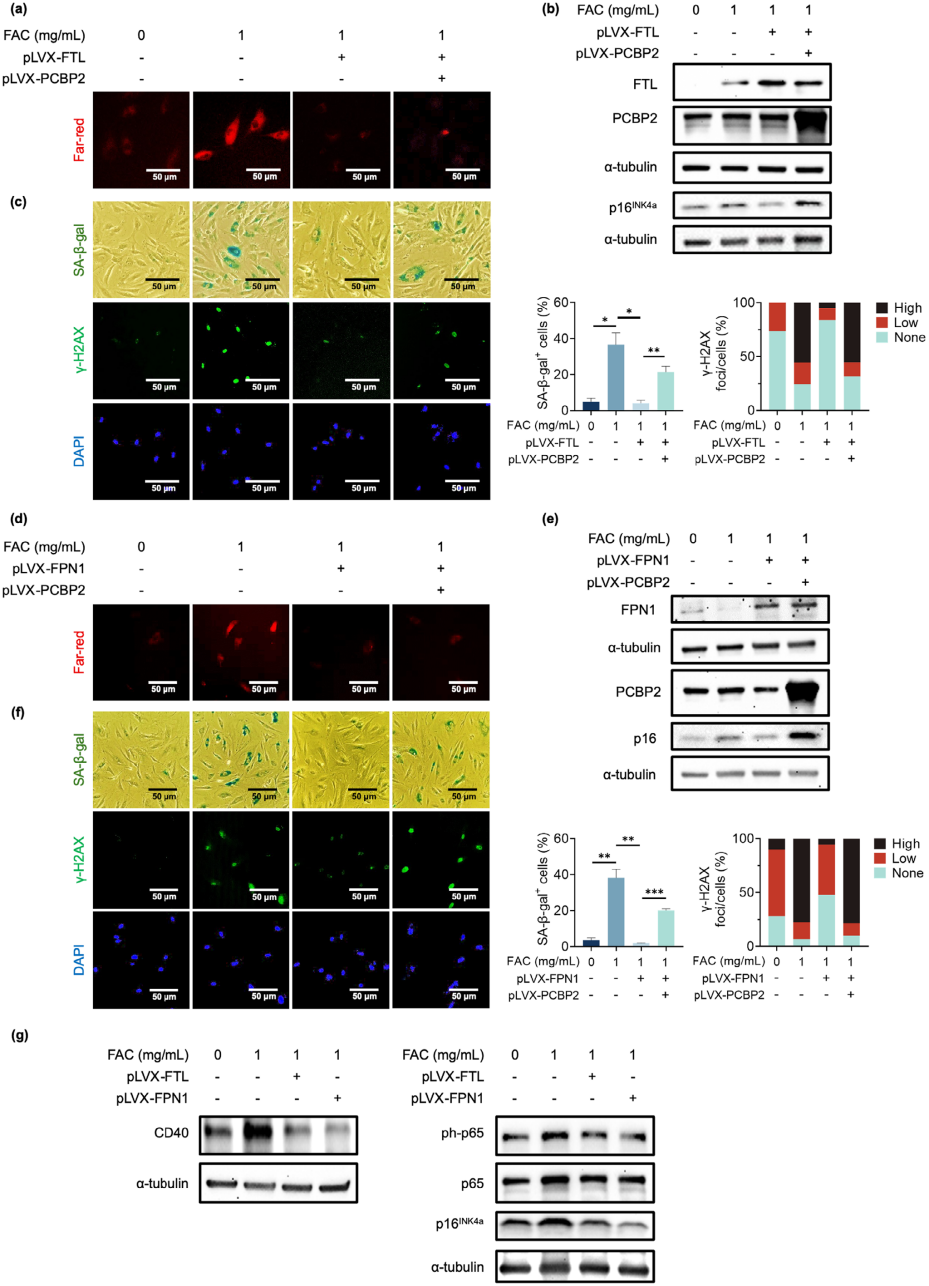
Overexpression of both ferritin and FPN1 suppresses the iron‐induced cellular senescence in HUVECs. (a) Far‐red labile Fe^2+^ staining shows that overexpression of FTL reduces the labile iron pool in the FAC‐treated p8 HUVECs, and overexpression of PCBP2 in these cells does not change the level of the labile iron. Data for far‐red labile Fe^2+^ staining represent *n* = 3 biologically independent experiments. (b) Western blots show that additional overexpression of FTL reduces the FAC‐induced upregulation of p16^INK4a^, and overexpression of PCBP2 recovers the expression of p16^INK4a^ in p8 HUVECs. Data for Western blots represent *n* = 3 biologically independent experiments. (c) SA‐β‐gal and γ‐H2AX staining show that overexpression of FTL rescues the FAC‐induced cellular senescence in p8 HUVECs, while additional overexpression of PCBP2 in these cells recovers the cellular senescence. DAPI stains the nuclei and serves as internal control. Quantitative plots (right) are shown for SA‐β‐gal‐positive cells (%) and γ‐H2AX foci/cells (%). Data represent *n* = 3 biologically independent samples. (d) Far‐red labile Fe^2+^ staining shows that overexpression of FPN1 reduces the labile iron pool in the FAC‐treated p8 HUVECs, and overexpression of PCBP2 in these cells does not change the level of the labile iron. Data for far‐red labile Fe^2+^ staining represent *n* = 3 biologically independent experiments. (e) Western blots show that overexpression of FPN1 reduces the FAC‐induced upregulation of p16^INK4a^ in p8 HUVECs and additional overexpression of PCBP2 in these cells recovers the expression of p16^INK4a^. Data for Western blots represent *n* = 3 biologically independent experiments. (f) SA‐β‐gal and γ‐H2AX staining show that overexpression of FPN1 rescues the FAC‐induced cellular senescence in p8 HUVECs, while additional overexpression of PCBP2 in these cells recovers the cellular senescence. DAPI stains the nuclei and serves as internal control. Quantitative plots (right) are shown for SA‐β‐gal‐positive cells (%) and γ‐H2AX foci/cells (%). Data represent *n* = 3 biologically independent samples. (g) Western blots showing that overexpression of FTL and FPN1 in FAC‐treated p8 HUVECs restored the expression of both CD40 and phosphorylation of NF‐κB p65 (ph‐p65) as well as p16^INK4a^ expression. FTL: Ferritin light chain; FPN1: Ferroportin 1; pLVX‐PCBP2: PCBP2 overexpression vector; pLVX‐FTL: FTL overexpression vector; and pLVX‐FPN1: FPN1 overexpression vector. *p‐value < 0.05; **p‐value < 0.01; ***p‐value < 0.001; ****p‐value < 1 × 10^−4^.

Iron is one of the essential elements for human life. However, an iron overload in the cell can induce ferroptosis (Yang and Stockwell 2016). To avoid this, cells employ at least three mechanisms to tightly control the intracellular iron levels (Kakhlon et al. 2001). Besides controlling iron uptake, overloaded iron in the cell can be either stored safely in ferritin, an iron cage that is made up of two functionally distinct proteins (FTH and FTL) (Garate and Nunez 2000; Kakhlon et al. 2001), or exported out of the cell by iron exporter FPN1 (Ganz 2005). Since accumulation of iron causes cellular senescence, as shown in Figure 6, we hypothesize that increased iron storage or its export could inhibit iron‐induced cellular senescence. To test this, far‐red labile Fe^2+^ staining (FerroFarRed) was performed on 1 mg/mL FAC‐treated ECs with or without FTL and FPN1 overexpression. While treating ECs with FAC significantly enhanced the far‐red labile Fe^2+^ staining, overexpression of both FTL and FPN1 substantially decreased the staining (Figure 7a,d, columns 1, 2, and 3). No obvious change in the staining was detected when PCBP2 was overexpressed in the FAC‐treated ECs with FTL and FPN1 overexpression (Figure 7a,d, column 4).

To test if ferritin and FPN1 regulate iron‐induced cellular senescence in ECs, we performed both SA‐β‐gal and γ‐H2AX staining in the ECs treated with either 1 mg/mL FAC, 1 mg/mL FAC plus FTL, or FPN1 overexpression, and 1 mg/mL FAC plus overexpression of PCBP2 in the FTL‐ or FPN1‐overexpressing ECs. Expression of FTL, FPN1, PCBP2, as well as of p16^INK4a^ in these cells was first confirmed by Western blot analysis. While FAC treatment expectedly upregulated the p16^INK4a^ expression as demonstrated in Figure 6, ECs treated with FAC also upregulated the FTL expression as previously reported (Aziz and Munro 1987). However, FPN1, as yet unknown, was downregulated (Figure 7b,e, lanes 1 and 2). Consistent with the decreased far‐red labile Fe^2+^ staining, overexpression of both FTL and FPN1 in the FAC‐treated ECs downregulated the expression of p16^INK4a^ (Figure 7b,e, lane 3). This decreased expression of p16^INK4a^ was reversed when PCBP2 was overexpressed (Figure 7b,e, lane 4). Moreover, consistent with the p16^INK4a^ expression profiles, overexpression of both FTL and FPN1 suppressed FAC‐induced cellular senescence (Figure 7c,f, columns 1, 2 and 3), and this suppression was reversed by overexpressing PCBP2 in the iron‐treated and FTL‐ or FPN1‐overexpressing ECs (Figure 7c,f, column 4).

In addition, our Western blots also demonstrated that overexpression of both FTL and FPN1 in the FAC‐treated ECs decreased the expression of CD40 and phosphorylated NF‐κB p65 in these cells as shown in Figure 7g.

Together, these data further demonstrate that iron regulates cellular senescence by upregulating p16^INK4a^ and CD40 via PCBP2.

## 3 Discussion

**FIGURE 8:**
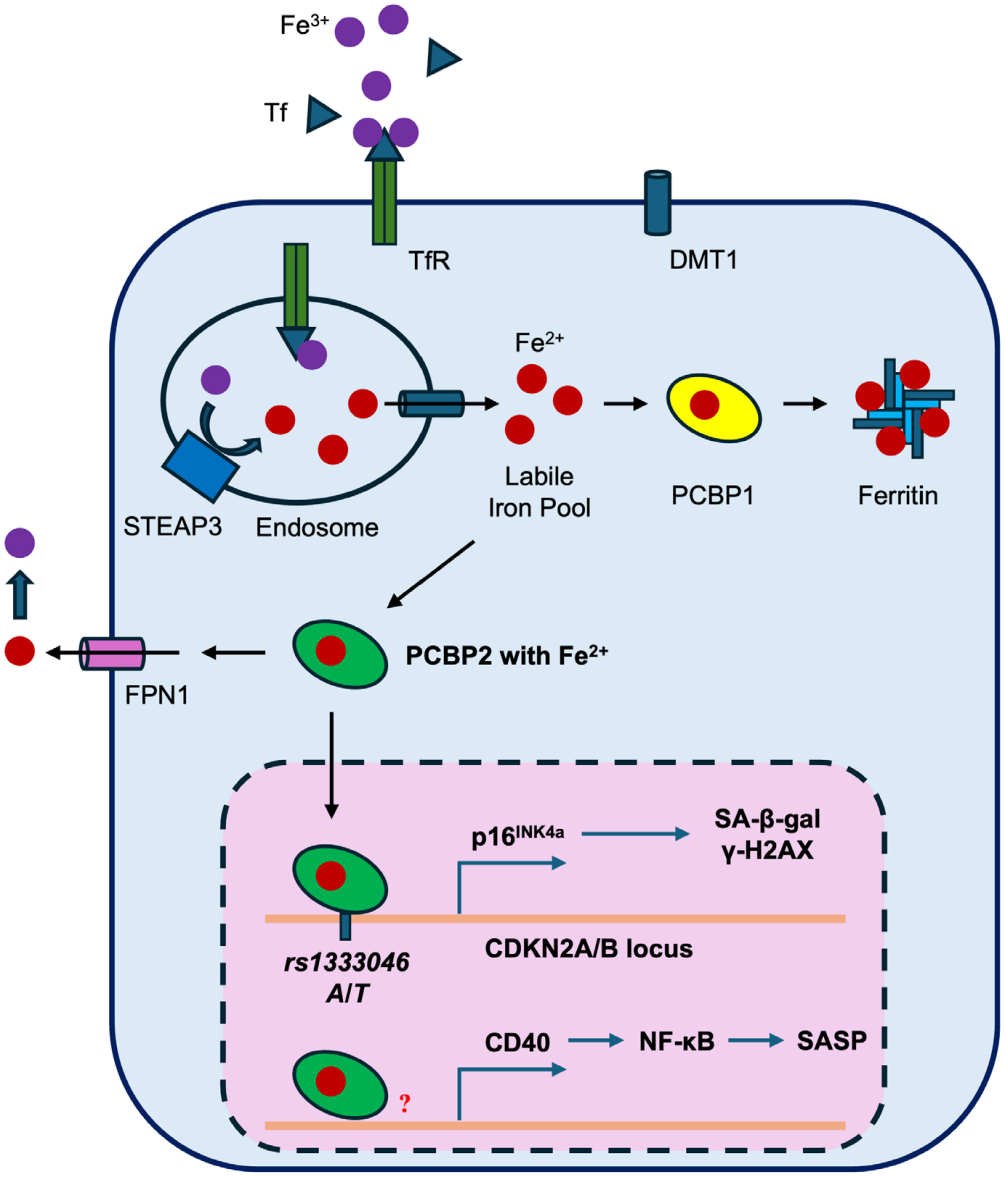
A simplified model shows how PCBP2 induces cellular senescence by modulating the expression of p16^INK4a^ and CD40 in response to iron. The lower panel of this figure shows that, at least in human ECs, PCBP2 binds to the atherosclerosis‐associated *rs1333046* on the CDKN2A/B locus, regulating cellular senescence by modulating the p16^INK4a^ expression. This will lead to upregulation of both SA‐β‐gal and γ‐H2AX. Moreover, PCBP2 also regulates CD40 expression, which is responsible for SASP gene activation via the CD40‐induced NF‐κB signaling pathway. However, it is currently not known how PCBP2 regulates CD40 expression. The upper panel shows that iron enters the cell via Tf‐TfR‐mediated endocytosis and forms a labile iron pool. The accumulated iron in the cytosol can either be stored in ferritin mediated by PCBP1 or exported out of the cell through FPN1 mediated by PCBP2, otherwise, overloaded iron can induce cellular senescence by upregulating both p16^INK4a^ and CD40. While upregulation of p16^INK4a^ is regulated by enhanced binding of PCBP2 to *rs1333046*, it is currently not known how PCBP2 regulates CD40. Consistent with this model, we show that overexpression of both FTL and FPN1 can suppress cellular senescence by reducing the intracellular labile iron pool. Tf, Transferrin; TfR, Tf receptor; DMT1, divalent metal transporter 1; STEAP3, Six‐transmembrane epithelial antigen 3.

Based on our post‐GWAS functional analysis, we demonstrated that PCBP2 is an activator of p16^INK4a^, inducing p16^INK4a^‐dependent endothelial senescence (Figure 8). This regulation depends on the allele‐imbalanced binding of PCBP2 to the fSNP *rs1333046*. We also discovered that PCBP2 regulates SASP gene expression via the CD40–NF‐κB signaling pathway. While these data are consistent with our knowledge that p16^INK4a^ does not regulate SASP genes (Coppe et al. 2011), we currently do not know how exactly PCBP2 regulates CD40 expression. Moreover, in our study, we also showed a passage‐dependent induction in the expression of p16^INK4a^, PCBP2, CD40, and phosphorylated NF‐κB p65, suggesting that these proteins are involved in regulating replicative senescence. Nevertheless, we believe that our findings provide a potential mechanism that explains the contribution of fSNP *rs1333046* as well as PCBP2 to the regulation of cellular senescence.

Consistent with our findings that PCBP2 regulates both p16^INK4a^ and CD40 transcription, PCBP2 was reported to be a transcription factor regulating gene transcription (Yuan et al. 2021). However, in contrast to our findings, recent studies reported that PCBP2 downregulation accelerated cellular senescence in mouse embryonic fibroblasts (MEFs) by inducing p73 mRNA instability and by reducing p73 expression (Ren et al. 2016). Therefore, more experiments are imperative to address the exact role of PCBP2 in regulating cellular senescence in different cell types.

We also realized that iron could induce endothelial senescence by activating both p16^INK4a^ and CD40‐mediated SASP gene expression through PCBP2, and that activation of p16^INK4a^ at least is dynamic, where the latter depends on the binding of PCBP2 to the fSNP *rs1333046*. To further demonstrate that iron is indeed an inducer of cellular senescence, we showed that overexpression of both the iron storage protein FTL and the iron exporter FPN1 inhibited iron‐induced endothelial senescence, as overexpression of these proteins can reduce the labile iron pool in the cell. Consistent with our findings, an increased labile iron pool was reported to induce cellular senescence albeit with different mechanisms, such as by regulating Robo4 expression (Noh et al. 2023) and by activating the cGAS‐STING pathway (Li et al. 2023). Thus, understanding how iron is regulated in vascular senescence may help us to identify a new therapeutic target in this disease.

Iron can also induce ferroptosis via the Fenton reaction (Winterbourn 1995). Both cellular senescence and ferroptosis are involved in the pathogenesis of age‐related diseases, such as neurodegeneration, cardiovascular disease, and cancer (Coradduzza et al. 2023). However, the underlying mechanisms that connect iron‐induced cellular senescence and ferroptosis are largely unknown. Based on our data, we now believe that, at least in ECs, iron can dynamically regulate the p16^INK4a^ expression. At low concentrations, iron induces endothelial senescence by activating p16^INK4a^ expression, and at high concentrations, iron suppresses p16^INK4a^ expression and induces ferroptosis by generating oxidized lipids. This raises the possibility of using ferroptosis as a senolytic to suppress cellular senescence. However, both cellular senescence and ferroptosis can be activated by different factors, where some can trigger both processes and others can only activate one process but not the other. Therefore, we think that the relationship between cellular senescence and ferroptosis is case‐dependent.

Collectively, in this report, we demonstrate a new mechanism underlying the contribution of PCBP2/*rs1333046* to atherosclerosis‐associated cellular senescence by modulating the p16^INK4a^ and CD40‐mediated SASP gene expression. We also demonstrate how iron, as an environmental factor, interacts with PCBP2/*rs1333046* to induce atherosclerosis‐associated cellular senescence. However, to fully understand the role that iron and PCBP2 play in regulating cellular senescence and atherosclerosis, a comprehensive in vivo functional study would be warranted.

## 4 Materials and Methods

### 4.1 Cell Culture and Reagents

Primary HUVECs (Cat#: PCS‐100‐013), 293 T (Cat#: CRL‐3216), and A549 (Cat#: CCL‐185) were purchased from ATCC. All cells are free of mycoplasma. HUVECs were cultured in Human EC Growth Medium V2 (Cell Applications, Cat#: 213–500). 293Ts and A549s were cultured in DMEM supplemented with 10% fetal bovine serum (FBS), 1% HEPES (1 M), and 1% glutamine. All cells were cultured at 37°C in a 5% CO_2_ incubator.

### 4.2 Primers and Antibodies

All DNA oligonucleotides used in this study were purchased from IDT and are listed in Table S1. All antibodies used are listed in Table S2 with their corresponding supplier information.

### 4.3 SDCP‐MS

SNP‐specific DNA competition pulldown—mass spectrometry (SDCP‐MS) was performed as described previously (Zhao et al. 2020). In brief, ~15 μg of the SDCP construct DNA was conjugated to 150 μL streptavidin‐coupled Dynabeads (Life Technologies, Cat#: 11206D) according to the manufacturer's instructions. The DNA‐beads were then washed and mixed with 1 mg nuclear extract (NE) in a buffer containing a 40‐fold excess of 7d competitor at RT for 1 h. After separation and washing, the protein‐DNA‐beads were subjected to 5 μL *Eco*RI (100 units/μL, NEB, Cat#: R0101M) digestion at 37°C for 30 min to remove the 3′‐DNA plus the proteins bound to this non‐SNP region. After separation and washing, the protein‐DNA‐beads were subsequently treated with 5 μL *Bam*HI (100 units/μL, NEB, Cat#: R3136M) at 37°C for 45 min to release the fSNP sequence plus the fSNP‐bound proteins. The supernatant was loaded onto an 8% short SDS‐PAGE gel and then collected for protein complex identification by mass spectrometry. For mass spectrometry analysis, two technical replicates were performed in parallel for each SNP.

To identify fSNP‐specific binding proteins, we first eliminated all the proteins with peptide counts in both samples and controls. We also eliminated all the proteins with peptide spectrum counts only in one of the two replicates for each fSNP. The fSNP‐specific bound proteins were identified as those proteins that have protein peptide spectrum counts in two replicates for one fSNP, but not in the two replicates for the controls performed in parallel.

### 4.4 EMSA

EMSA was performed using the LightShift Chemiluminescent EMSA Kit (Thermo Fisher Scientific, Cat#: 20148) according to the manufacturer's instructions. Probes of both alleles were generated by annealing two 31‐bp SNP‐centered oligos, one of which contained 3′‐biotinylation. NE was isolated from HUVECs. Probes and NE were mixed and incubated at RT for 30 min, followed by electrophoresis on a 6% TBE native gel and a mobility shift assay. Data represent *n* = 3 independent biological replicates.

### 4.5 Luciferase Reporter Assay

Luciferase reporter assays were performed in 293 T cells using the pGL3‐Promoter vector (Promega, Cat#: E1761). 31‐bp SNP‐centered fragments were cloned using the *Sac*I and *Xho*I sites into the pGL3‐Promoter vector. The same amounts of both SNP allele constructs were transfected into 293 T by FuGENE HD transfection reagent (Promega, Cat#: E2311). Additionally, we transfected the same amounts of an internal control vector, pRL‐TK (Promega, Cat#: E2241), which constitutively expressed the *Renilla* luciferase. Relative luciferase reporter activities were measured by normalization of firefly luciferase activities to *Renilla* luciferase activities, using the Dual‐Glo Luciferase Assay System (Promega, Cat#: E2920). All experiments followed the manufacturer's protocol. Data represent *n* = 6 independent biological replicates.

### 4.6 ChIP Assay

ChIP assays were performed as described earlier (Noss et al. 2015). HUVECs were fixed with 1% formaldehyde for 10 min and quenched by 0.14 M glycine. Sonication was carried out at 37% amplitude with cycles of 20 s on and 50 s off for 3 min, and subsequently the supernatants of the mixture were incubated overnight with 5 μg of anti‐PCBP2 antibody coupled to Dynabeads Protein A/G (Thermo Fisher Scientific, Cat#: 10001D & 10003D) after spinning down. DNA pulled down by antibody and input DNA were purified with Zymoclean Gel DNA Recovery Kit (Zymo Research, Cat#: D4007). Purified DNAs were then used for real‐time qPCR analysis for the DNA fragments containing fSNP *rs1333046* with primers 1333046_PCR_F and 1333046_PCR_R (see Table S1). ChIP assay results were measured by normalization of ChIP DNA quantity to input DNA. For antibody isotype specificity control, a rabbit anti‐IgG antibody was used. Data represent *n* = 3 independent samples.

### 4.7 AIDP‐Wb Analysis

AIDP‐Wb analyses were performed as described earlier (Zhao et al. 2020). In brief, a 31‐bp biotinylated SNP‐centered DNA fragment containing each allele was generated by annealing two DNA oligonucleotides. Approximately 1 μg of DNA was then attached to 50 μL of Dynabeads M‐280 Streptavidin (Thermo Fisher Scientific, Cat#: 11206D). DNA beads were then mixed with ~500 μg of NE isolated from HUVECs at 4°C overnight.

with rotation. After washing off unbound proteins, the DNA‐bound proteins were eluted using Western blot sampling buffer solution and separated on SDS‐PAGE gel for immunoblot analyses using protein‐specific antibodies. As an internal loading control, the same blot was probed with the PARP‐1‐specific antibody. Data represent *n* = 3 independent samples.

### 4.8 RT‐qPCR Analysis

Total RNA was isolated using the RNeasy Mini Kit (Qiagen, Cat#: 74106). Complementary DNA (cDNA) was then synthesized with the iScript cDNA Synthesis Kit (Bio‐Rad, Cat#: 1708890) after treatment with DNase I (Thermo Fisher Scientific, Cat#: EN0521). All procedures followed the manufacturers' protocols. qPCR was performed with the QuantStudio 5 Real‐Time PCR System (Applied Biosystems) according to the protocol for either SYBR Green PCR Master Mix (Applied Biosystems, Cat#: 4364344) or TaqMan Universal PCR Master Mix (Applied Biosystems, Cat#: 4304437). The following primer mixes for TaqMan qPCR were purchased from Applied Biosystems: *p14* ^*ARF*^ Hs99999189_m1; *p15* ^*INK4b*^ Hs00793225_m1; *p16* ^*INK4a*^ Hs02902543_mH; ANRIL Hs04259472_m1; *IL‐1B* Hs01555410_m1; and *GAPDH* internal control Hs02786624_g1. Other primers used in SYBR green qPCR are listed in Table S1. Data represent *n* = 3 independent samples.

### 4.9 Western Blot Analysis

Whole‐cell proteins were isolated with RIPA buffer (Sigma, Cat#: R0278). Proteins were separated on SDS‐PAGE gel and transferred to nitrocellulose membranes. Proteins were detected using protein‐specific antibodies. All antibodies purchased and used are listed in Table S2. For internal loading control, α‐tubulin was used. Data represent *n* = 3 independent samples. The data obtained from these Western blots are quantified by densitometry analysis as presented in Figures S3 and S4.

### 4.10 SA‐β‐Gal Staining

The Senescence β‐Galactosidase Staining Kit (Cell Signaling, Cat#: 9860S) was used to visualize senescent cells. Visualization was performed using an RVL‐100‐G microscope (Echo Laboratories). Images were analyzed with ImageJ (v.1.52K, NIH). Data represent *n* = 3 independent biological replicates.

### 4.11 γ‐H2AX Staining

For the γ‐H2AX staining, HUVECs seeded in 96‐well plates were fixed with 4% paraformaldehyde, and the cell membrane was solubilized using DPBS containing 1% BSA, 0.1% Tween‐20 and 0.2% Triton X‐100. Cells were incubated with anti‐H2AX (phosphorylated H2A histone family member X) antibody in the solubilizing solution overnight. Then, the immunofluorescence staining was detected with Alexa Fluor 488‐conjugated antibody. Visualization was done with an RVL‐100‐G microscope (Echo Laboratories). Images were analyzed using ImageJ software (v.1.52K, NIH). The intensity is defined as “high” when the staining covered the whole nuclei, “low” when there were only dots, and “none” when no staining was detected. Data represent *n* = 3 independent biological replicates.

### 4.12 BrdU Incorporation Assay

HUVEC proliferation was determined using the BrdU incorporation assay following the protocol of the BrdU Cell Proliferation Assay Kit (Cell Signaling, Cat#: 6813). Briefly, HUVECs were first treated with lentiviruses for 48 h, and then transferred (10,000 cells per well) to 96‐well plates for 24 h. 1× BrdU was added to the culture medium for DNA labeling. The labeling medium was removed after 2 h, then cells were fixed, and DNA was denatured by adding 100 μL fixing/denaturing solution for 30 min. The incorporated BrdU was then detected by a mouse anti‐BrdU monoclonal antibody and measured using an anti‐mouse IgG conjugated with HRP. Data for the BrdU proliferation assay represent *n* = 8 independent biological samples.

### 4.13 siRNA Knockdown

The siRNA for CD40 was purchased from IDT. The internal control siRNA was purchased from Ambion (Cat#: 4390803). Transfection was performed following the protocol of Lipofectamine RNAiMAX Transfection Reagent (Invitrogen, Cat#: 13778075). Forty‐eight hours after transfection, cells were collected for different assays. The target sequences for siRNAs are listed in Table S1.

### 4.14 Overexpression of PCBP2, p16^INK4a^ , and CD40

For overexpression of PCBP2 and p16^INK4a^, the cDNAs of human PCBP2 and p16^INK4a^ were cloned into the lentiviral vector pLVX‐M‐puro using *Xho*I and *Eco*RI cutting sites. The CD40 overexpression vector was purchased from VectorBuilder. Lentiviruses were generated by transfection of 293 T cells together with psPAX2 and pMD2.G plasmids.

### 4.15 Far‐Red Labile Iron Staining

The far‐red labile iron staining was performed according to the protocol provided by the manufacturer (Millipore, Cat#: SCT037). Cells, which had been treated with lentiviruses and FAC, were rinsed with DPBS. Following a 1.5‐h incubation of BioTracker Far‐red Labile Fe^2+^ live cell dye in serum‐free medium, the cells were rinsed with and observed in serum‐free medium. Visualization was done with an RVL‐100‐G microscope (Echo Laboratories). Images were analyzed using ImageJ software (v.1.52 K, NIH). Data represent *n* = 3 independent biological replicates.

### 4.16 Immunocytochemical Staining

Human atherosclerotic plaque samples were obtained from patients who underwent carotid endarterectomy. The part of the carotid artery showing hard, calcified tissue was used as a plaque zone while the part far from the unaffected zone was used as the normal zone. Both plaque and normal‐appearing zones were separated and fixed in 4% buffered formalin for 2 h and stored in 30% sucrose solution containing 0.05% sodium azide overnight. Sections of 10‐μm thickness were permeabilized with 0.1% Triton X‐100 for 4 h and blocked overnight in PBS containing 2% BSA in 96‐well cell culture plates. Sections were incubated for a further 24 h with primary antibodies against PCBP2 (Abcam, Cat#: ab184962, 1:250 dilution). After washing with PBS, sections were incubated for 1 h at RT with fluorochrome‐conjugated secondary antibodies (Alexa Fluor 488 goat anti‐mouse and Alexa Fluor 647 goat anti‐rabbit). Tissue sections were stained and mounted with VECTASHIELD DAPI, and images were taken using confocal laser microscopy and analyzed with ImageJ (v.1.52 K, NIH). Data represent two independent experiments, with *n* = 5 plaque zones and *n* = 5 normal‐appearing zones.

### 4.17 Statistical Analysis

All normally distributed data are presented as mean ± s.e. *p*‐values were calculated using the two‐tailed Student's *t*‐test. Non‐normally distributed data relating to quantification of *PCBP2* immunocytochemical staining in Figure 3 are presented as median ± interquartile range, and *p* values were calculated with the nonparametric Mann–Whitney test for pairwise comparison. Multiple‐group data of FAC‐treatment ChIP assay in Figure 6i were analyzed by single‐factor ANOVA. All data presented in this work are reproducible. No statistical method was used to predetermine sample size. No data were excluded from the analyses. The experiments were not randomized, and no blinding was applied to allocation during experiments.

## Author Contributions

G.L. designed the study, analyzed the data, and drafted and revised the manuscript; S.Y. performed all the experiments and participated in drafting the manuscript; Y.W. performed the SDCP‐MS; L.L. and Z.W. assisted with EMSA, AIDP‐Wb, and shRNA knockdown; E.J. and P.D. assisted with immunostaining.

## Conflicts of Interest

The authors declare no conflicts of interest.

## Supporting information


**Figure S1:** Western blots showing that PCBP2 is an activator of p15^INK4b^ and a suppressor of p14^ARF^. (a) and (b) showing that shRNA‐mediated PCBP2 knockdown (shPCBP2) resulted in downregulation of p15 and upregulation of p14. (c) and (d) showing that ectopic cDNA overexpression of PCBP2 (pLVX‐PCBP2) upregulates p15 and downregulates p14. shScrambled: control for shRNA knockdown; pLVX‐puro: control vector for PCBP2 overexpression.
**Figure S2:** C11‐BODIPY staining shows that FAC can induce oxidized lipids at concentrations above 1 mg/mL. ECs were treated with the indicated concentrations of FAC and stained with C11‐BODIPY performed using the BODIPY 581/591 C11 Kit (Invitrogen, catalog no. D3861). After washing with 3xPBS, cells were mounted using Fluoromount‐G Mounting Medium with DAPI (Invitrogen catalog no. 00‐4959‐52). Cells were visualized using a confocal microscope. Images were quantified using the ImageJ software to calculate the relative levels of lipid peroxidation, based on the ratio of oxidized fluorescence to non‐oxidized fluorescence signals.
**Figure S3:** Densitometry analysis for Figures 1d, 2a,c,m, 4a,f, 5a,b,e,g,j.
**Figure S4:** Densitometry analysis for Figures 6a,f,j, 7b,e,g.
**Table S1:** Primers used in this study.
**Table S2:** Antibodies used in this study.

## Data Availability

All the data are included in the manuscript and Supplementary Figures and also available at request.
