# Supplementary material for: PCBP2 Regulates p16INK4a ‐Dependent Cellular Senescence in Response to Iron
Source: Aging Cell. 2025 Nov 11;24(12):e70283. doi: 10.1111/acel.70283 (PMC12686567; doi:10.1111/acel.70283)
Supplement: Supplementary file 1 — Figure S1: Western blots showing that PCBP2 is an activator of p15INK4b and a suppressor of p14ARF. (a) and (b) showing that shRNA‐mediated PCBP2 knockdown (shPCBP2) resulted in downregulation of p15 and upregulation of p14. (c) and (d) showing that ectopic cDNA overexpression of PCBP2 (pLVX‐PCBP2) upregulates p15 and downregulates p14. shScrambled: control for shRNA knockdown; pLVX‐puro: control vector for PCBP2 overexpression. Figure S2: C11‐BODIPY staining shows that FAC can induce oxidized lipids at concentrations above 1 mg/mL. ECs were treated with the indicated concentrations of FAC and stained with C11‐BODIPY performed using the BODIPY 581/591 C11 Kit (Invitrogen, catalog no. D3861). After washing with 3xPBS, cells were mounted using Fluoromount‐G Mounting Medium with DAPI (Invitrogen catalog no. 00‐4959‐52). Cells were visualized using a confocal microscope. Images were quantified using the ImageJ software to calculate the relative levels of lipid peroxidation, based on the ratio of oxidized fluorescence to non‐oxidized fluorescence signals. Figure S3: Densitometry analysis for Figures 1d, 2a,c,m, 4a,f, 5a,b,e,g,j. Figure S4: Densitometry analysis for Figures 6a,f,j, 7b,e,g. Table S1: Primers used in this study. Table S2: Antibodies used in this study. [file ACEL-24-e70283-s001.docx]

**Supplementary Figure 1. Western blots showing that PCBP2 is an activator of p15^INK4b^ and a suppressor of p14^ARF^. (a)** and **(b)** showing that shRNA-mediated PCBP2 knockdown (shPCBP2) resulted in downregulation of p15 and upregulation of p14. **(c)** and **(d)** showing that ectopic cDNA overexpression of PCBP2 (pLVX-PCBP2) upregulates p15 and downregulates p14. shScrambled: control for shRNA knockdown; pLVX-puro: control vector for PCBP2 overexpression.

**
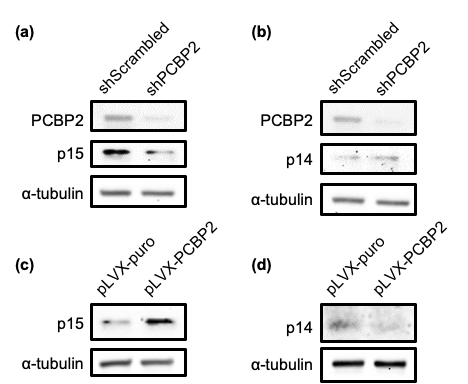
**

**Supplementary Figure 2. C11-BODIPY staining shows that FAC can induce oxidized lipids at concentrations above 1 mg/mL.** ECs were treated with the indicated concentrations of FAC and stained with C11-BODIPY performed using the BODIPY™ 581/591 C11 Kit (Invitrogen, catalog no. D3861). After washing with 3xPBS, cells were mounted using Fluoromount-G™ Mounting Medium with DAPI (Invitrogen catalog no. 00-4959-52). Cells were visualized using a confocal microscope. Images were quantified using the ImageJ software to calculate the relative levels of lipid peroxidation, based on the ratio of oxidized fluorescence to non-oxidized fluorescence signals.

**
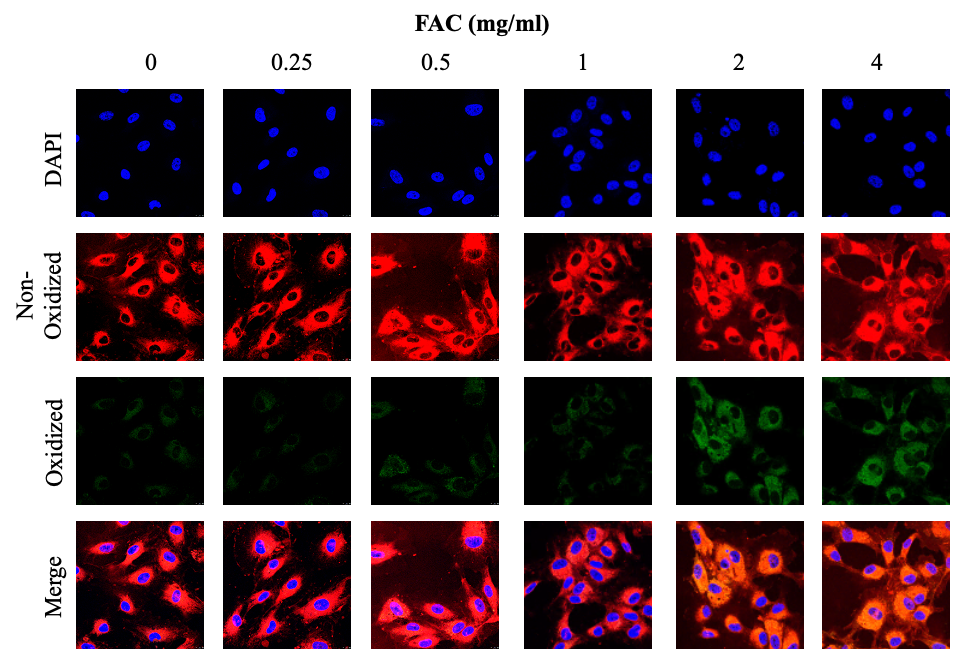
**

**Supplementary Figure 3.** Densitometry analysis for Fig. 1d, Fig. 2a, Fig. 2c, Fig. 2m, Fig. 4a, Fig. 4f, Fig. 5a, Fig. 5b, Fig. 5e, Fig. 5g and Fig. 5j.


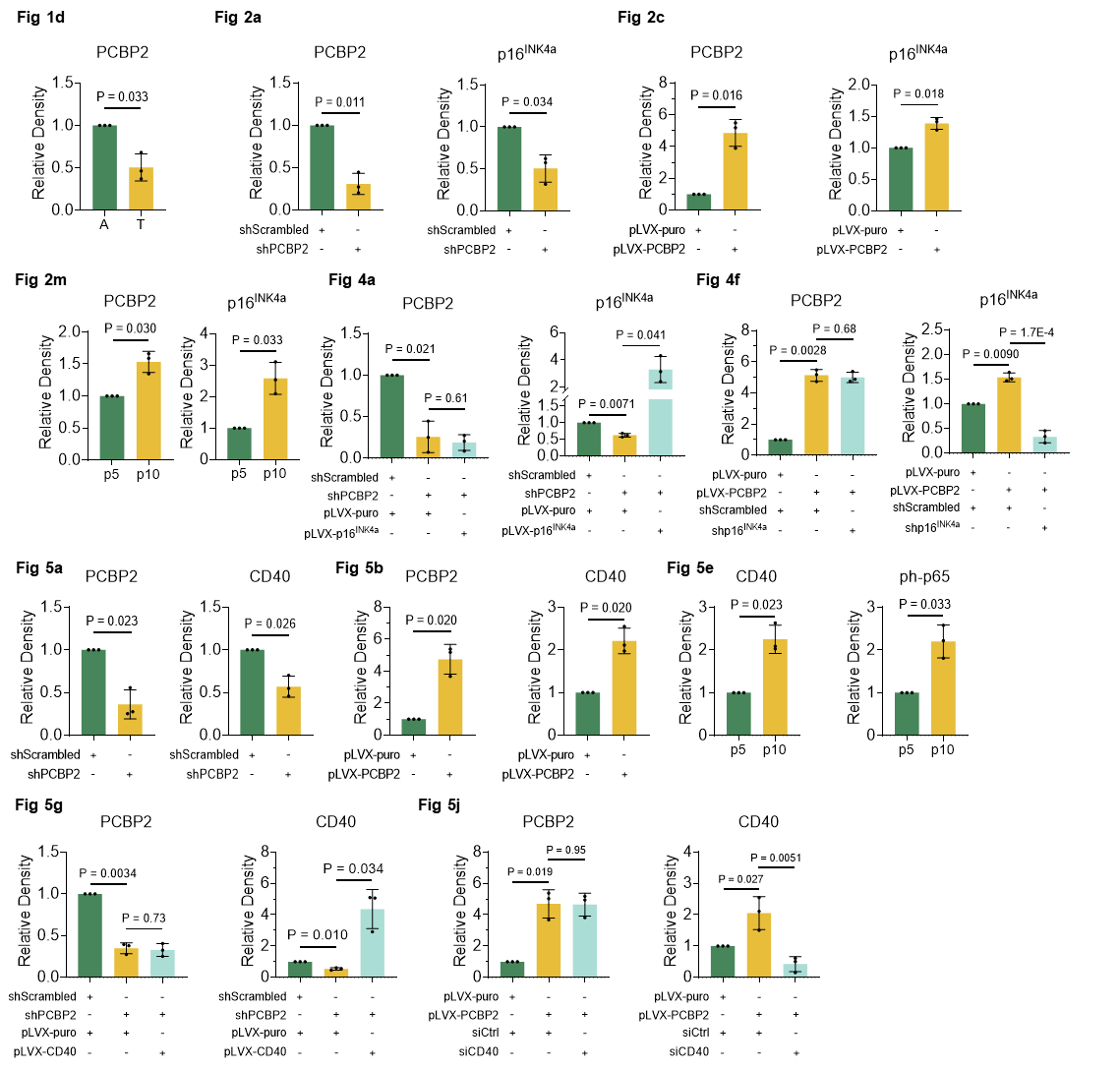


**Supplementary Figure 4.** Densitometry analysis for Fig. 6a, Fig. 6f, Fig. 6j, Fig. 7b, Fig. 7e and Fig. 7g.

**
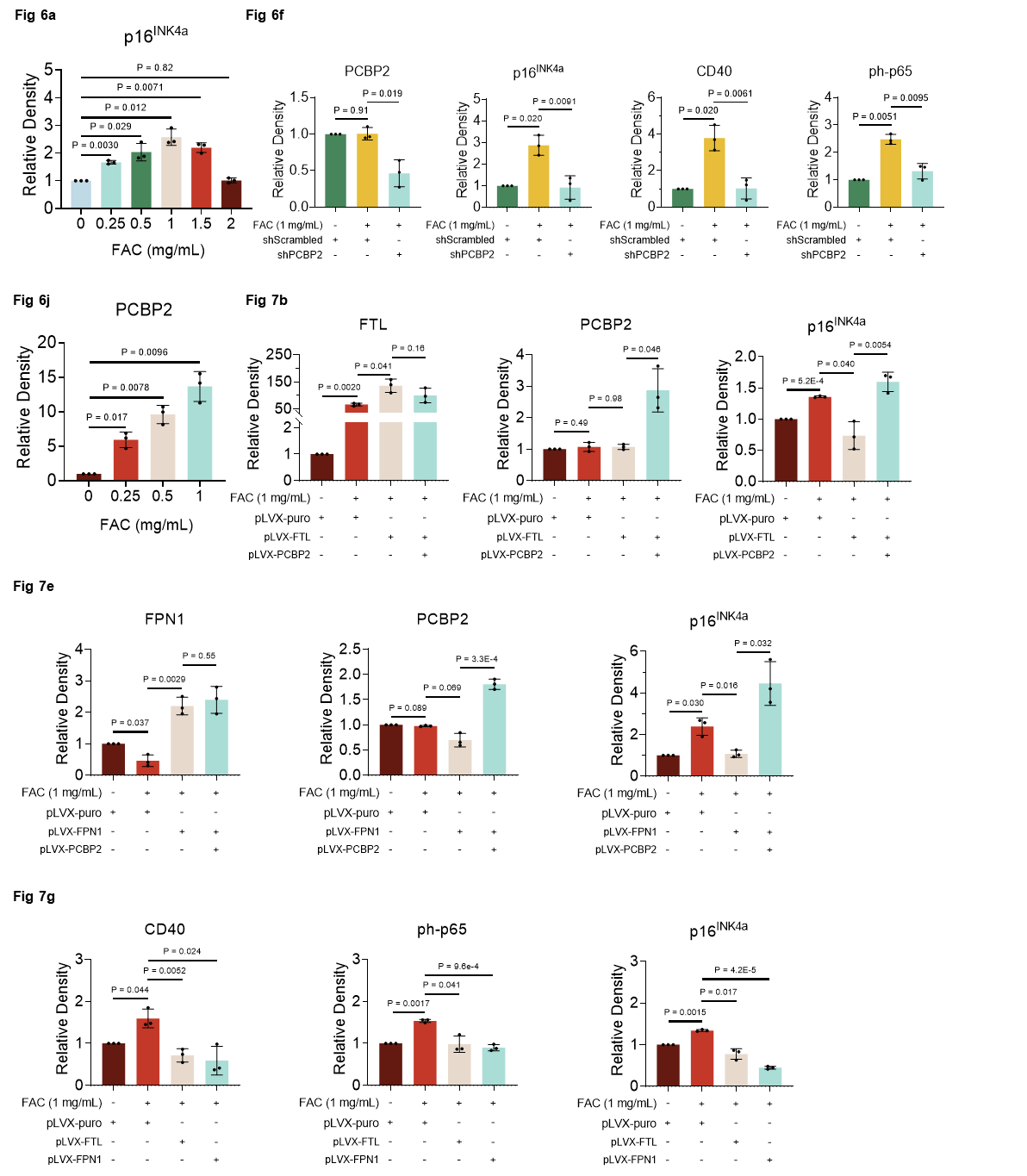
**

**Supplementary Table 1. Primers used in this study.**

| Usage | Name | Sequence |
| --- | --- | --- |
| qPCR | PCBP2-F | tctggggcgcagatcaaaat |
|  | PCBP2-R | gtctccgaggaaagcctgac |
|  | IL6-F | GCAGAAAACAACCTGAACCTT |
|  | IL6-R | ACCTCAAACTCCAAAAGACCA |
|  | ICAM1-F | ACCTCAAACTCCAAAAGACCA |
|  | ICAM1-R | TCTGAGACCTCTGGCTTCGTCA |
|  | GAPDH-F | CGACCACTTTGTCAAGCTCA |
|  | GAPDH-R | AGGGGTCTACATGGCAACTG |
|  | CD40-F | actgatgttgtctgtggtcccc |
|  | CD40-R | tggcttcttggccacctttttg |
| shRNA knockdown | PCBP2 (1083-1103) | AAGCTGCACCAGTTGGCAATG |
| SDCP | SDCP-bio046A-F | GTCTGTGTTCCGTTGTCCGTGCTGAATGGATCCGGATCCAAACAATTTAGAACAATTGGCAATATATATAGAATTCGAATTCGC |
|  | SDCP-bio046A-R | GCGAATTCGAATTCTATATATATTGCCAATTGTTCTAAATTGTTTGGATCCGGATCCATT |
|  | SDCP-046A-F-7D | GTCTGTGTTCCGTTGTCCGTGCTGAATGGATCCGGATCCAAACAATTTAGAGCAATATATATAGAATTCGAATTCGC |
|  | SDCP-046A-R-7D | GCGAATTCGAATTCTATATATATTGCTCTAAATTGTTTGGATCCGGATCCATT |
| Luciferase Reporter Assay | 046A-LRA insert | AAACAATTTAGAACAaTTGGCAATATATATA |
|  | 046T-LRA insert | AAACAATTTAGAACAtTTGGCAATATATATA |
| ChIP | 1333046_F | CATATGCATAGACAAATACACC |
|  | 1333046_R | TTTAGTTGCGACCTCAGAAAG |
| siRNA target | CD40 | agagaaaaacagtaccta |

**Supplementary Table 2. Antibodies used in this study.**

| **Antibody** | **Cat#** | **Usage** | **Amount** | **Link** |
| --- | --- | --- | --- | --- |
| PCBP2 | sc-101136 | WB, ChIP | 1:500 | https://www.scbt.com/p/hnrnp-e2-antibody-23-g |
| PARP1 | sc-8007 | WB | 1:10000 | https://www.scbt.com/p/parp-1-antibody-f-2 |
| p16^INK4A^ | 10883-1-AP | WB | 1:500 | https://www.ptglab.com/products/P16,P19-Antibody-10883-1-AP.htm#product-information |
| p15^INK4B^ | PA5-49749 | WB | 1:500 | https://www.thermofisher.com/antibody/product/CDKN2B-Antibody-Polyclonal/PA5-49749 |
| p14^ARF^ | sc-53639 | WB | 1:500 | https://www.scbt.com/p/p14-arf-antibody-dcs-240 |
| γ-H2AX | sc-517348 | IF | 1:50 | https://www.scbt.com/p/p-histone-h2a-x-antibody-ser-139 |
| α-tubulin | 66031-1-Ig | WB | 1:10000 | https://www.ptglab.com/products/tubulin-Alpha-Antibody-66031-1-Ig.htm |
| CD40 | A2018 | WB | 1:500 | https://abclonal.com/catalog-antibodies/CD40RabbitpAb/A0218 |
| Endogenous NF-κB p65 | 8242 | WB | 1:500 | https://www.cellsignal.com/products/primary-antibodies/nf-kb-p65-d14e12-xp-174-rabbit-mab/8242 |
| Phospho-NF-κB p65 S536 | 3033T | WB | 1:500 | https://www.cellsignal.com/products/primary-antibodies/phospho-nf-kb%02p65-ser536-93h1-rabbit-mab/3033?site-search-type=Products |
| Ferritin light chain | sc-74513 | WB | 1:500 | https://www.scbt.com/p/ferritin-light-chain-antibody-d-9 |
| Ferroportin 1 | 26601-1-AP | WB | 1:500 | https://www.ptglab.com/products/SLC40A1-Antibody-26601-1-AP.htm |
| Rabbit anti-IgG | 2729 | ChIP | 5 uL | https://www.cellsignal.com/products/primary%02antibodies/normal%02rabbit%02igg/2729 |
| m-IgG Fc BP-HRP | sc-525409 | WB | 1:10000 | https://www.scbt.com/p/m-igg-fc-bp-hrp |
| mouse anti-rabbit IgG-HRP | sc-2357 | WB | 1:10000 | https://www.scbt.com/p/mouse-anti-rabbit-igg-hrp |
